# Advancing Torsades de pointes risk prediction: unveiling the role of drug metabolites through molecular docking

**DOI:** 10.1093/toxres/tfaf186

**Published:** 2026-01-29

**Authors:** Egemen Bilgin, Gulcin Tugcu, Ahmet Aydin

**Affiliations:** Yeditepe University, Faculty of Pharmacy, Department of Toxicology, Kayışdağı, Istanbul 34755, Türkiye; Yeditepe University, Faculty of Pharmacy, Department of Toxicology, Kayışdağı, Istanbul 34755, Türkiye; Yeditepe University, Faculty of Pharmacy, Department of Toxicology, Kayışdağı, Istanbul 34755, Türkiye

**Keywords:** Torsades de pointes, human ether-à-go-go-related gene, cardiotoxicity, In silico risk assessment, molecular docking

## Abstract

This study explores the risk of Torsades de Pointes (TdP) arrhythmia, focusing on the interactions of parent drugs and their metabolites with the Human ether-à-go-go-related gene (hERG) channel, which is crucial in cardiac electrical activity and TdP risk assessment. Using a dual-strategy molecular docking approach with AutoDock Vina and PatchDock, we analyzed clinically relevant ligand pairs: astemizole/desmethylastemizole, terfenadine/fexofenadine, and quetiapine/norquetiapine. Quantitative analysis revealed that high binding affinity does not always correlate with toxicity. For instance, the non-cardiotoxic metabolite fexofenadine exhibited a higher binding affinity (−9.3 kcal/mol) compared to its toxic parent terfenadine (−8.9 kcal/mol), but its safety is explained by physicochemical constraints (zwitterionic nature). Conversely, desmethylastemizole maintained high affinity (−9.2 kcal/mol) with a geometrically “relaxed” fit (Atomic Contact Energy: −338.36), rationalizing its sustained potency. Geometric analysis further distinguished quetiapine as a “steric blocker” (Contact Area: ~588 Å^2^) causing forced occlusion, whereas its metabolite norquetiapine acted as a specific ligand with a significantly smaller interface area (~417 Å^2^). These findings highlight the importance of focusing not only on the parent drug but also on metabolites for TdP risk assessment in new drug development. We advocate for an integrated computational framework combining binding energy, geometric complementarity, and physicochemical profiling to enhance the accuracy of early cardiac safety screenings.

## Introduction

Although drug-induced arrhythmias have received increased attention recently, they have been a persistent safety concern for decades. Initially, TdP cases in the 1970s and 1980s were primarily associated with antiarrhythmic agents. This perspective shifted dramatically in 1990 when it was discovered that non-cardiac drugs, such as the widely used antihistamine terfenadine, could also induce QT prolongation and TdP.^1^ Subsequently, other non-antiarrhythmic drugs—including another antihistamine, astemizole; the gastrokinetic cisapride; the antibiotic erythromycin; opiates such as levomethadyl and methadone; and the lipid-lowering agent probucol—were linked to TdP risk.^2^^,^^3^ The recognition that TdP could occur across diverse therapeutic classes led to the market withdrawal of several high-profile medications.^4^

Consequently, in the 1990s, regulatory bodies such as the European Medicines Agency (EMA) and the U.S. Food and Drug Administration (FDA) mandated routine preclinical and clinical testing for QT-prolongation potential in new drug candidates.^5^ Non-clinical (International Council for Harmonisation [ICH] S7A and S7B) and clinical (ICH E14) guidelines were established to define the requisite studies for assessing QT interval prolongation risk. However, a prolonged QT interval is not always a definitive indicator of TdP risk.^6^ Sanguinetti et al. highlighted the mechanistic link between QT prolongation and the inhibition of voltage-dependent K+ channels, particularly the rapid delayed rectifier current (IKr), which is essential for ventricular repolarization. It was further noted that the molecular interactions with this channel are primarily associated with hERG, the pore-forming subunit of the channel.^7^

Recent developments in cardiac safety assessment emphasize the predominance of in vitro evaluation of hERG channel blockage.^8^ However, concurrent risk factors such as age, gender, electrolyte imbalance, bradycardia, and pre-existing structural heart disease significantly influence TdP incidence. Notably, TdP events may involve interactions across multiple ion channels, proarrhythmic mechanisms independent of hERG, and the specific effects of metabolites or the antiarrhythmic properties of the parent drug. These complexities underscore the inadequacy of relying solely on surrogate markers or the parent compound alone to accurately estimate TdP risks. Therefore, a more comprehensive evaluation that considers the collective effects of all active agents—including both parent drugs and their metabolites—is recommended for effective safety assessment.^9^

In this study, we evaluated three clinically relevant pairs: astemizole/desmethylastemizole, quetiapine/norquetiapine, and terfenadine/fexofenadine. These pairs were selected due to the documented differences in torsadogenic potential between the metabolites and their parent compounds, representing clinically relevant examples of hERG-related cardiotoxicity. For astemizole, the desmethyl metabolite circulates at higher concentrations than the parent and exhibits similar potency toward hERG, likely contributing substantially to arrhythmogenic risk.^10–12^ In the case of quetiapine, while the parent blocks the hERG potassium current, its primary metabolite, norquetiapine, affects the Nav1.5 sodium current, potentially mitigating QT prolongation driven by hERG suppression.^13^ For terfenadine, the parent inhibits hERG, whereas its active metabolite, fexofenadine, does not, a distinction that underpinned the approval of fexofenadine and the subsequent discontinuation of terfenadine.^14–16^

To address these complexities, we employed an integrated in silico approach using AutoDock Tools (ADT), AutoDock Vina, PatchDock, and BIOVIA Discovery Studio Visualizer to assess the interactions of these parent-metabolite ligand pairs with the hERG channel. By combining docking scores with rigorous post-docking geometric analysis, we advocate for a holistic approach to TdP risk assessment. This strategy aims to improve the prediction accuracy of TdP risk, optimize resource utilization, and accelerate the translation of computational models into clinical safety evaluations for new drugs.

## Materials and methods

**Fig. 1:**
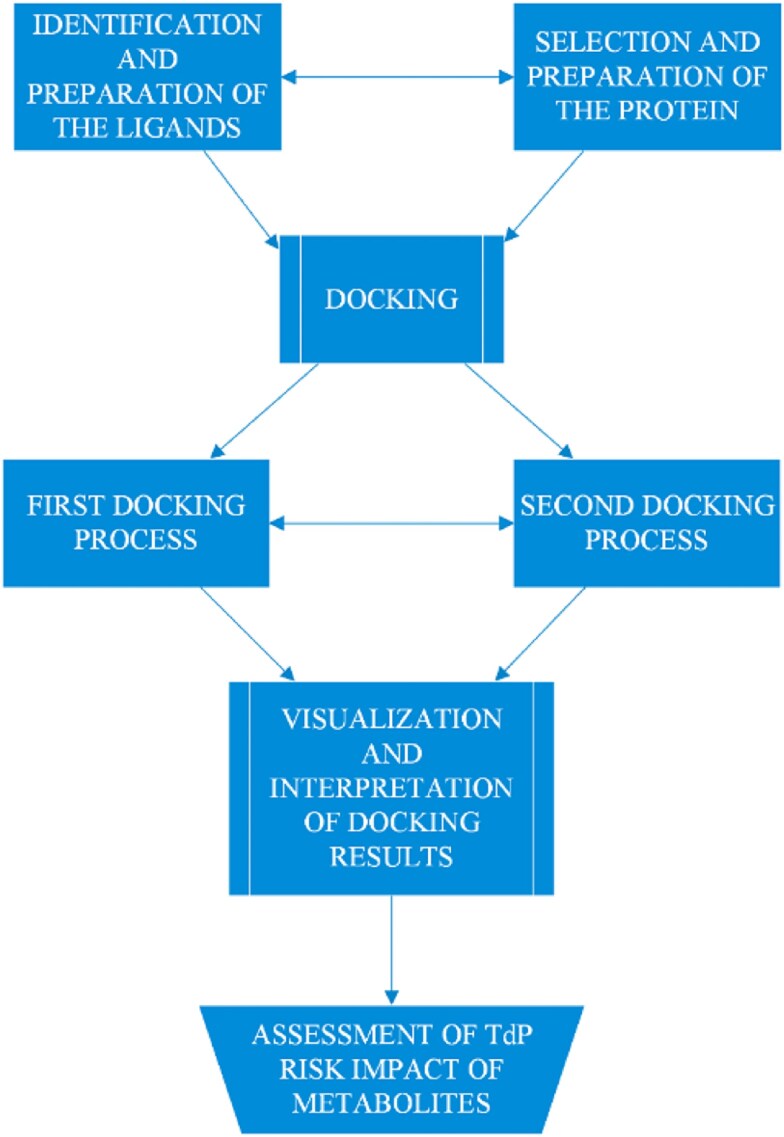
Overview of the study approach.

Our study relies primarily on molecular docking techniques to assess how metabolites might impact the torsadogenic potential of their parent molecules. The overall approach used in this study is illustrated in Fig. 1.

### Molecular docking

In this study, we implemented two complementary docking strategies. The first strategy was conducted using AutoDock Vina and BIOVIA Discovery Studio Visualizer. In the second strategy, PatchDock was utilized instead of AutoDock Vina and was used in conjunction with BIOVIA Discovery Studio Visualizer.

To leverage the strengths of these two distinct tools, AutoDock Vina was used to estimate binding energy (how strongly a molecule binds to a protein) and binding conformation (how the molecule aligns within the protein’s binding site). In contrast, PatchDock focuses on geometric compatibility between interacting molecules. Thus, PatchDock is primarily concerned with shape alignment, whereas AutoDock Vina aims to identify the optimal binding pose by evaluating energy levels on a grid. Although both methods evaluate molecular surfaces, they emphasize different aspects of the interaction.^17–20^ This dual-strategy approach facilitates a comparative assessment of how complementary information from both tools informs TdP risk. In evaluating the docking results, we adopted a comparative approach rather than relying on a single absolute threshold. However, binding energies lower (more negative) than −7.0 kcal/mol were generally considered indicative of significant binding potential, consistent with typical hit identification thresholds in virtual screening campaigns. A complete list of tools used in the study is provided in Table 1.

**Table 1:** List of tools and their purposes.

Name of tool	Purpose of usage
ADT Version 1.5.7	Preparation of ligands Preparation of the protein
AutoDock Vina	Scoring of ligand binding affinities to the protein
BIOVIA Discovery Studio Visualizer	Preparation of the protein Visualization of ligand-protein interactions
PatchDock	Scoring of geometric shape complementarity of ligand-protein complexes
PROCHECK Tool	Evaluation of the stereochemical quality of the prepared protein structure
OpenBabel	Addition of explicit hydrogens Correction of the protonation state at pH 7.4 Conversion of SDF into PDB format (for Autodock Vina)
PrankWeb Tool	Active site identification of the protein

#### The first docking process

ADT Version 1.5.7, AutoDock Vina and BIOVIA Discovery Studio Visualizer were used to perform the first docking process.^19–22^ The primary objective was to determine the binding affinities of the ligands to the hERG protein.

##### Selection and preparation of the protein

Molecular docking was performed using a protein structure retrieved from Protein Data Bank (PDB) at http://www.rcsb.org. Details of the three-dimensional Cryo-EM structure of the K + -bound hERG channel are listed in Table 2.

**Table 2:** Details of Cryo-EM structure of K + -bound hERG channel.

Protein	PDB ID	PDB DOl	EMDB Entry	Classification	Structure
K + -bound hERG channel	7CN1	https://doi.org/10.2210/pdb7CN1/pdb	EMD-30413 (3.7 Å)	Transport Protein	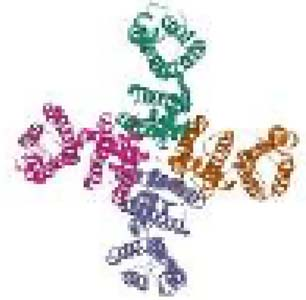

The hERG structure (PDB ID 7CN1) was refined by using BIOVIA Discovery Studio Visualizer. The protein structure was obtained in PDB format and imported into BIOVIA Discovery Studio Visualizer. Protein preparation in BIOVIA Discovery Studio Visualizer included the removal of all heteroatoms, bound ligands, and crystallographic water molecules, followed by the addition of hydrogen atoms to satisfy valences. Heteroatoms were removed to clear the active site for subsequent protein-ligand interaction calculations. Polar hydrogens were then added using BIOVIA Discovery Studio Visualizer to facilitate the evaluation of hydrogen-bonding interactions during docking. Subsequently, Kollman United Atom partial charges were assigned to the protein using ADT. In accordance with the united-atom representation standard for AutoDock protein preparation, nonpolar hydrogens were merged; adding only polar hydrogens and assigning Kollman charges was deemed sufficient. The final prepared protein structure is shown in Supplementary Fig. S1.

##### Validation of the quality of prepared protein

A Ramachandran plot analysis was performed to assess the quality of the prepared protein. This analysis is essential for confirming the validity of the protein PDB file that is subsequently used for docking. The PROCHECK tool was used to evaluate the stereochemical quality of the protein structure, represented through a Ramachandran plot (Supplementary Fig. S2).^23^ Based on the validation results, all residues were within the preferred and allowed regions of the plot (Supplementary Fig. S3).

##### Active site identification of the prepared protein

Active sites were identified using the PrankWeb tool (http://prankweb.cz/), which provides a web interface enabling users to easily carry out the prediction and visually inspect the predicted binding sites via an integrated sequence-structure view. The sequence view comprises predicted pockets, computed conservation and binding sites (if present in the PDB file). PrankWeb reports pockets with probability scores; residues with higher scores are more likely to be involved in binding interactions.^24–26^

##### Preparation of ligands for docking

Ligand 3D structures were retrieved from PubChem as SDF files via the Compound Summary download options; these conformers are computational rather than experimental. Using Open Babel, explicit hydrogens were added (−h) and protonation states were adjusted to physiological pH (7.4) with the -p option, which applies atom-by-atom protonation.^27^ Since tautomer/ionization choices can influence docking, we fixed the protomer at pH 7.4 and retained the tautomer present in the PubChem SDF without tautomer enumeration, noting this as a limitation. Structures were then converted to PDB with Open Babel, and the final ligand preparation in ADT included computing Gasteiger partial charges, merging non-polar hydrogens, defining rotatable bonds (torsion tree/TORSDOF), and exporting as PDBQT for docking.

##### Further details of the first docking process

BIOVIA Discovery Studio Visualizer was used to analyze ligand-protein interactions. AutoDock Vina, a molecular-dynamics-inspired, simulated-annealing-based docking algorithm, was used to score interacting compounds.

AutoDock Vina uses a grid-based representation of protein-ligand interaction potentials to calculate the binding affinity, which helps predict ligand-protein interactions and aids in the design of potential ligands. It uses soft-core potentials to effectively generate multiple conformations of small molecules and macromolecules within the target binding site.

#### The second docking process

PatchDock is another docking tool used in this study. It accepts two molecules as input, which can be proteins, DNA, peptides, or drugs. The output is a ranked list of possible complexes based on shape complementarity. PatchDock is primarily a web-based tool accessible at https://bio3d.cs.huji.ac.il/webserver/patchdock.^17^^,^^18^

##### Selection and preparation of the protein

The same protein used in the first docking process was also employed in the second (PatchDock) workflow. For PatchDock, the protein structure in PDB format retrieved from the Protein Data Bank was uploaded to the PatchDock web interface as the protein input.

##### Active site identification of the protein

The same active sites identified with PrankWeb for the first docking process were used here. In PatchDock, these are referred to as protein binding sites. The sites were listed in a text document and uploaded to the PatchDock system along with the PDB files.

##### Preparation of ligands for the second docking

Each ligand used in the first docking process was also employed in the second (PatchDock) workflow. Ligand 3D structures were retrieved from PubChem as SDF (computational 3D conformers) via the Compound Summary download options. Using Open Babel, explicit hydrogens were added (−h) and protonation states were standardized to physiological pH 7.4 with the -p option, which applies atom-by-atom protonation. The resulting pH 7.4 was fixed and the PubChem tautomer was retained without enumeration (limitation noted). The standardized ligands were then converted to PDB format with Open Babel for PatchDock, which accepts PDB inputs; accordingly, ligands were docked in their predominant ionization state at pH 7.4.

##### Further details of second docking process

To initiate docking, the required inputs were submitted to the PatchDock server. An email address was provided to enable delivery of the results. Upon completion, PatchDock sent a link via email for convenient access to the results. As in the first docking process, BIOVIA Discovery Studio Visualizer was used to examine ligand-protein interactions.

## Results

**Fig. 2:**
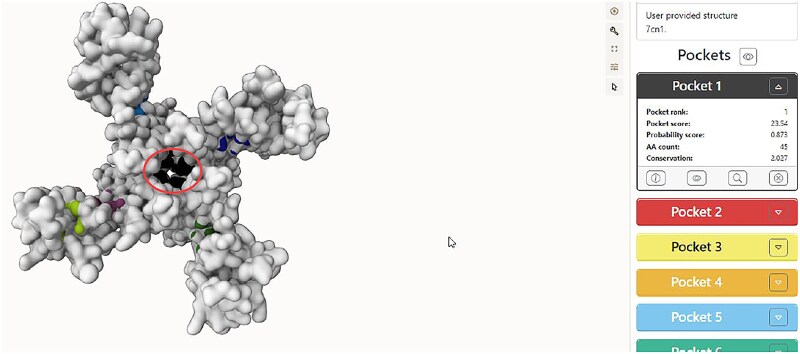
PrankWeb prediction of the binding site of the prepared protein.

After uploading the prepared protein to the respective tool, PrankWeb provided 17 available pockets with their probability scores. Pocket 1 (Fig. 2) which had the highest probability score of 0.873, was chosen. Structurally, this pocket corresponds to the canonical drug-binding site of the hERG central pore, encompassing key aromatic residues such as TYR652 and PHE656, which are established determinants of high-affinity drug blockade. The area marked with a red ring in Fig. 2 represents Pocket 1. This pocket contained the following residues: A_622 A_623 A_624 A_625 A_648 A_649 A_652 A_653 A_656 A_660 B_621 B_622 B_623 B_624 B_625 B_645 B_648 B_649 B_652 B_653 B_656 B_660 C_621 C_622 C_623 C_624 C_625 C_645 C_648 C_649 C_652 C_653 C_656 C_657 D_621 D_622 D_623 D_624 D_645 D_648 D_649 D_652 D_653 D_656 D_657.

**Fig. 3:**
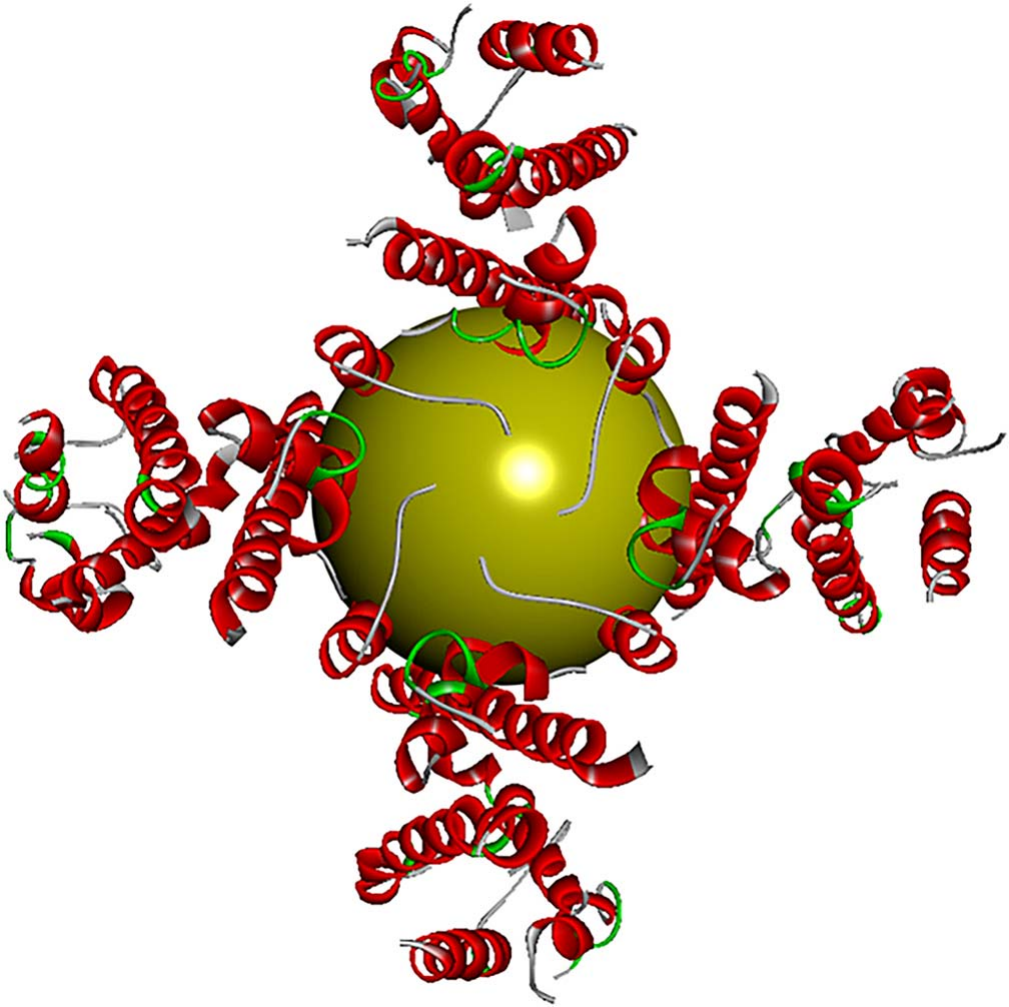
Visualization of the binding sites of the prepared protein.

Following the identification of possible binding sites, the specific residues listed above were selected within the protein structure using BIOVIA Discovery Studio Visualizer to determine the x, y, and z coordinates of the docking site. This step is crucial because the docking system places the ligands based on these coordinates. To achieve this, the residues in Pocket 1 were selected across the four chains (A–D), and the software was used to define the docking region. The defined binding site, formed by the selected high-probability residues (represented by a yellow sphere), is shown in Fig. 3.

**Fig. 4:**
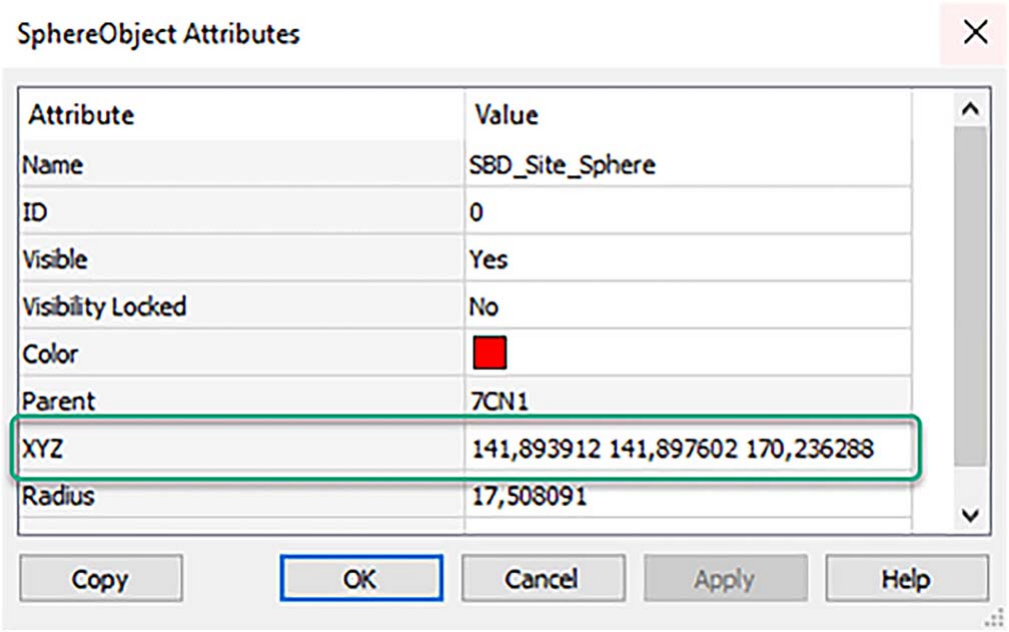
X, Y and Z coordinates of the binding site for the protein.

The numerical coordinates of the binding site center were determined using the same software. BIOVIA Discovery Studio Visualizer displayed the x, y, and z coordinates of this specified area, as shown in Fig. 4.

After these steps, the selected protein was prepared in ADT Version 1.5.7 prior to docking. The following parent-metabolite pairs were used for docking to the hERG protein:

- astemizole and desmethylastemizole
- quetiapine and norquetiapine
- terfenadine and fexofenadine

### Astemizole and desmethylastemizole

#### Results of the first docking process

##### Results from AutoDock Vina

AutoDock Vina indicated a modest affinity advantage for desmethylastemizole over astemizole in the top-scoring mode (Mode 1): −9.2 vs. −8.8 kcal/mol, respectively (Table 3). Complete AutoDock Vina outputs for both ligands are found in Supplementary Figs. S4 and S5.

**Table 3:** Binding scores of astemizole and desmethylastemizole.

Parent/Metabolite	Affinity (kcal/mol)
Astemizole	−8.8
Desmethylastemizole	−9.2

##### Results from BIOVIA discovery studio visualizer

**Fig. 5:**
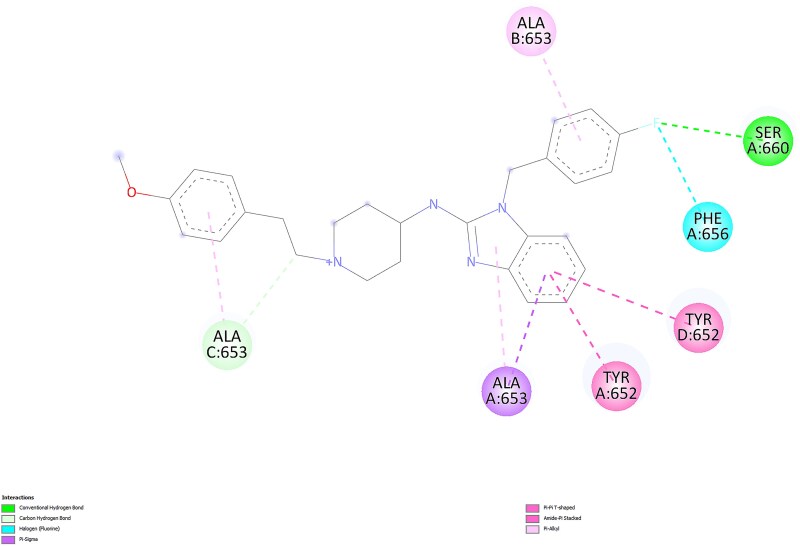
2D interactions of astemizole with the protein in the most favorable conformation.

**Fig. 6:**
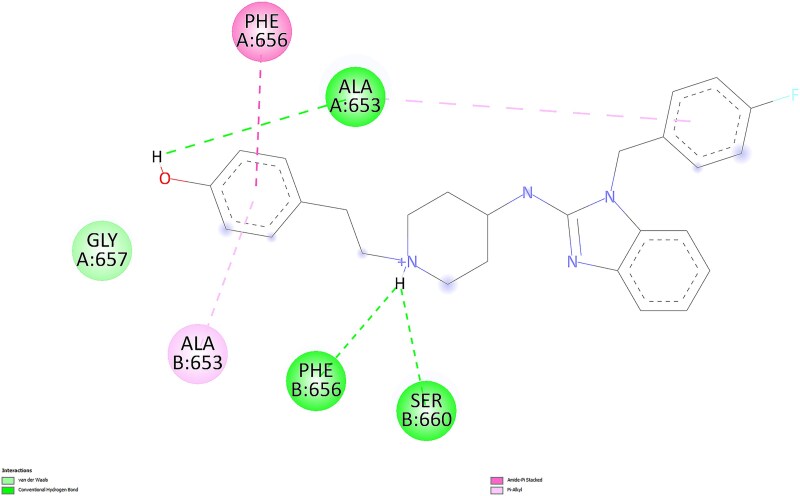
2D interactions of desmethylastemizole with the protein in the most favorable conformation.

For the most favorable conformations, ligand-protein interactions for astemizole and desmethylastemizole are presented in 2D in Figs. 5 and 6, respectively, as visualized using BIOVIA Discovery Studio Visualizer.

The astemizole pose engages the hERG binding pocket through π interactions with TYR 652 (chains A and D) and ALA 653 (chain A), together with hydrophobic/π–alkyl contacts around the central aromatic system. Notably, the para-fluorinated ring forms a halogen contact toward PHE 656 and a weak contact in the vicinity of SER 660, while additional carbon–hydrogen contacts are observed with ALA 653 (chain C). Overall, the interaction pattern is dominated by aromatic and hydrophobic contacts, with limited conventional hydrogen bonding.

In contrast, the desmethylastemizole pose shows added polar engagement consistent with its phenolic OH and protonated amine. Conventional hydrogen bonds are observed from the phenolic OH (e.g. toward ALA 653, chain A) and from the protonated tertiary amine toward SER 660 and PHE 656 (chain B). These hydrogen bonds complement π/π–alkyl contacts to PHE 656 and ALA 653 (chain A), together with van der Waals contacts near GLY 657. The presence of multiple, well-placed hydrogen bonds in the metabolite’s pose, alongside comparable aromatic contacts, provides a plausible structural rationale for its slightly stronger Vina score and suggests improved geometric compatibility within the pocket. Therefore, to contextualize the energy-based findings and better understand geometric compatibility and pocket engagement, we incorporated a second docking strategy focused on shape complementarity and steric effects.

#### Results of the second docking process

##### Results from PatchDock and BIOVIA discovery studio visualizer

By default, PatchDock provides 30 solutions. In our study, the top 10 solutions exhibiting the best geometric shape complementarity were further analyzed via BIOVIA Discovery Studio Visualizer to better reveal the details of the ligand-protein interactions. The top 10 solutions for astemizole and desmethylastemizole, along with their PatchDock metrics, are shown in Tables 4 and 5, respectively. Complete PatchDock results regarding the top 10 solutions, including the type of interactions for both ligands (2D diagrams), are found in Supplementary Figs. S6 and S7.

**Table 4:** PatchDock metrics of astemizole with score, area and ACE.

Solution number	Score	Area	ACE
1	6,146	718.9	−326.61
2	5,660	604.9	−230.08
3	5,428	607.2	−252.1
4	5,096	706.6	−325.86
5	5,002	564.5	−240.33
6	4,974	647.8	−294.69
7	4,940	619.1	−246.19
8	4,884	602.2	−208.12
9	4,806	543.7	−216.64
10	4,778	612.6	−285.89

**Table 5:** PatchDock metrics of desmethylastemizole with score, area and ACE.

Solution number	Score	Area	ACE
1	5,360	601.8	−253.52
2	5,354	699.6	−338.36
3	5,348	591.7	−246.49
4	5,318	707.1	−343.31
5	5,188	631.3	−261.35
6	5,086	568.9	−228.92
7	5,048	577.3	−235.42
8	5,014	557.9	−234.2
9	4,960	652.5	−287.21
10	4,958	681.1	−334.6

##### Interpretation of PatchDock metrics

- Score & Area: Astemizole yields the highest numerical Score (6146) and Area (718.9 Å^2^) in its top solution, suggesting a very extensive surface engagement. Desmethylastemizole also shows large contact areas (reaching ~707 Å^2^) but generally presents slightly lower geometric scores compared to the parent compound’s peak values.
- ACE (Atomic Contact Energy): A critical divergence is observed in energetics. While astemizole has some favorable poses, many of its top solutions exhibit shallower ACE values (e.g. −230.08, −208.12), indicating less optimal atomic packing. In contrast, desmethylastemizole achieves deeply negative ACE values (≤ −330) across multiple high-Area solutions (e.g. poses 2, 4, and 10), reflecting a more energetically favorable and well-packed interface.

##### Astemizole – Unfavorable-bump readout from 2D diagrams

- Incidence: Astemizole shows a high frequency of steric clashes, with 8 out of 10 poses displaying at least one unfavorable bump.
- Frequently clashing residues: The clash map is widely distributed across the binding pocket, involving TYR 652 (chains B/D/A), SER 660, SER 624, ALA 653, GLY 657, and PHE 656.
- Link to Metrics: This broad distribution of steric stress explains the shallower ACE values observed in Table 4. Although the molecule covers a large area (High Score/Area), it does so at the cost of significant steric conflict (“forced fit”), which energetically penalizes the binding event.

##### Desmethylastemizole – Unfavorable-bump readout from 2D diagrams

- Incidence: The metabolite displays a reduced bump incidence, with only 6 out of 10 poses showing bumps, and notably, several poses are completely bump-free.
- Frequently clashing residues: Clashes are more concentrated, mainly involving TYR 652 and SER 624, without the diffuse steric stress seen in the parent compound.
- Link to Metrics: The presence of multiple clean, bump-free poses correlates with the highly negative ACE values observed. Desmethylastemizole is able to achieve extensive contact (High Area) while maintaining a relaxed and well-packed geometry (Deep ACE), unlike the strained engagement of astemizole.

##### Consistency with ACE + area

- Astemizole: The combination of the highest geometric Score but frequent bumps and shallower ACE values points to a binding mode characterized by widespread steric interference. The molecule occupies the pore extensively but struggles to find an energetically relaxed conformation due to clashes with critical rim residues like TYR 652 and PHE 656.
- Desmethylastemizole: The metabolite successfully combines large Interface Areas with deeply negative ACE values (Table 5) and a lower incidence of bumps. This consistency suggests that desmethylastemizole can accommodate itself within the hERG pocket more effectively than astemizole, achieving a tight and stable blockade without the extensive steric penalties incurred by the parent drug.

The geometric docking results provide a structural explanation for the high inhibitory potential of desmethylastemizole compared to its parent compound. While astemizole engages the pore region extensively (high Area), it does so under significant steric conflict (“forced fit”), characterized by frequent bumps against the pore walls. This steric stress may energetically compromise the stability of the binding event.

In contrast, desmethylastemizole achieves a comparable pore occupancy but with a fundamentally different geometric quality. The lack of extensive steric clashes and the deeply negative ACE values indicate that the metabolite fits into the binding pocket with a “relaxed and stable geometry.” This structural compatibility implies that desmethylastemizole can maintain a tight and prolonged blockade of the ion pathway without the destabilizing steric penalties observed with astemizole. Consequently, the metabolite’s ability to “snugly” occupy the pore region—combined with its higher binding affinity (−9.2 vs −8.8 kcal/mol)—supports the rationale that desmethylastemizole acts as a highly potent and stable hERG inhibitor.

### Quetiapine and norquetiapine

#### Results of the first docking process

##### Results from AutoDock Vina

AutoDock Vina indicated a modest affinity advantage for norquetiapine over quetiapine in the top-scoring mode (Mode 1): −7.7 vs. −7.3 kcal/mol, respectively (Table 6). Complete AutoDock Vina outputs for both ligands are found in Supplementary Figs. S8 and S9.

**Table 6:** Binding scores of quetiapine and norquetiapine.

Parent / Metabolite	Affinity (kcal/mol)
Quetiapine	−7.3
Norquetiapine	−7.7

##### Results from BIOVIA discovery studio visualizer

**Fig. 7:**
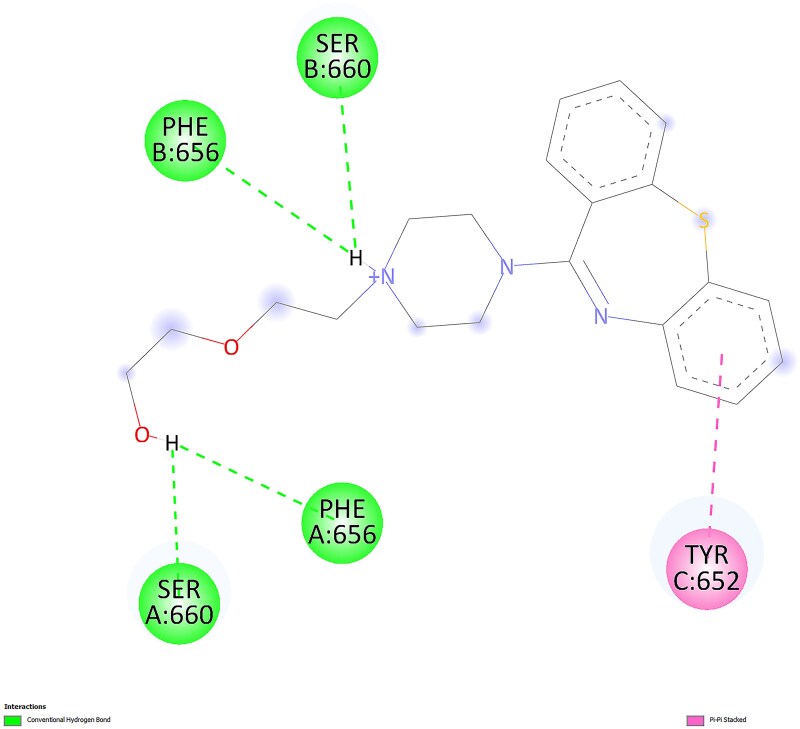
2D interactions of quetiapine with the protein in the most favorable conformation.

**Fig. 8:**
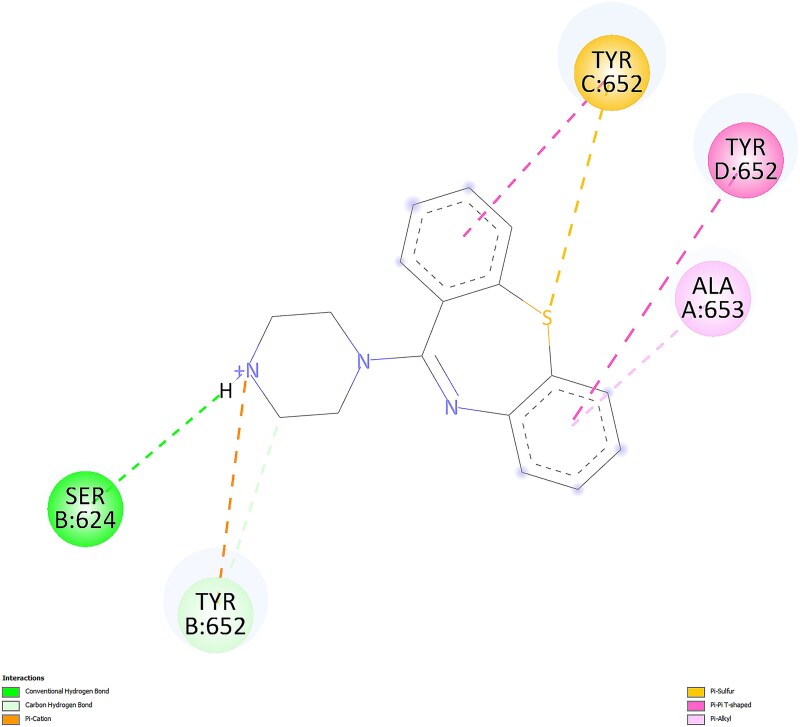
2D interactions of norquetiapine with the protein in the most favorable conformation.

For the most favorable conformations, ligand-protein interactions for quetiapine and norquetiapine are presented in 2D in Figs. 7 and 8, respectively, as visualized using BIOVIA Discovery Studio Visualizer.

Quetiapine pose engages the hERG binding pocket with a mixed polar–aromatic profile. Conventional hydrogen bonds are observed (e.g. from the protonated amine and the terminal alcohol) to nearby residues, while the dibenzothiazepine ring system participates in π–π stacking with TYR 652. Overall, the pose combines several hydrogen bonds with π interactions in the Y652 region.

In contrast, norquetiapine pose preserves similar aromatic engagement in the Y652 region and introduces additional polar and electrostatic features. Specifically, the protonated amine forms a conventional hydrogen bond with a nearby serine and engages in a cation–π interaction to TYR 652; the thioether ring contributes a π–sulfur contact to Y652, and the terminal ring shows π–π T-shaped/π–alkyl contacts to residues lining the pocket. This added combination of conventional hydrogen bonding, cation–π, and π–sulfur interactions, alongside comparable aromatic contacts, provides a plausible structural rationale for the slightly stronger Vina score of the metabolite and suggests improved geometric compatibility within the pocket. Taken together, the Mode 1 visuals indicate that norquetiapine retains the aromatic/π network seen for quetiapine while introducing and/or strengthening polar and cation–π engagements around the Y652 region.

However, evidence from the literature clearly indicates that quetiapine inhibits hERG, whereas norquetiapine may act in the opposite direction to reduce the risk of QT prolongation caused by quetiapine-related hERG inhibition. Therefore, to contextualize the energy-based findings and better understand geometric compatibility and pocket engagement, we incorporated a second docking strategy focused on shape complementarity and steric effects.

#### Results of the second docking process

##### Results from PatchDock and BIOVIA discovery studio visualizer

To complement the affinity-based findings from AutoDock Vina, a geometric docking study was performed using PatchDock to evaluate shape complementarity, contact surface area, and steric compatibility within the hERG binding pocket. The top 10 solutions of quetiapine and norquetiapine with PatchDock metrics are shown in Tables 7 and 8 respectively. Complete PatchDock results regarding top 10 solutions including the type of interactions for both ligands (2D diagrams) are found in Supplementary Figs. S10 and S11.

**Table 7:** PatchDock metrics of quetiapine with score, area, and ACE.

Solution number	Score	Area	ACE
1	4,554	576.3	−273.28
2	4,496	511.2	−240.95
3	4,460	513.8	−252.69
4	4,142	518.3	−292.36
5	4,072	472.9	−249.29
6	4,060	509.3	−226.49
7	4,032	588.8	−289.87
8	4,006	441.7	−213.8
9	3,920	576.1	−291.18
10	3,910	469.8	−241.62

**Table 8:** PatchDock metrics of norquetiapine with score, area, and ACE.

Solution number	Score	Area	ACE
1	3,812	417.5	−192.68
2	3,722	454.6	−226.59
3	3,628	419.5	−181.8
4	3,486	468.1	−217.06
5	3,354	363.3	−203.88
6	3,272	357.2	−164.78
7	3,268	345.3	−198.61
8	3,250	504.0	−246.45
9	3,222	330.4	−192.0
10	3,192	357.2	−197.83

##### Interpretation of PatchDock metrics

- Score (Geometric Shape Complementarity): Quetiapine consistently yields higher geometric scores (Top solution: 4554) compared to norquetiapine (Top solution: 3812). This substantial difference of approximately 740 score units quantitatively supports the steric disparity, indicating that the bulkier parent molecule occludes the hERG pore cavity significantly more effectively than its metabolite.
- Area: The contact surface area for quetiapine is notably larger across the top solutions (e.g. 576.3 Å^2^ and 588.8 Å^2^) compared to norquetiapine, which generally presents smaller interface areas (e.g. 417.5 Å^2^). A larger area correlates with greater steric occlusion of the channel pore.
- ACE: While quetiapine shows highly negative ACE values driven by its large surface contact, norquetiapine exhibits modest ACE values, consistent with its smaller molecular footprint and specific, rather than broad, surface engagement.

##### Quetiapine – Unfavorable-bump readout from 2D diagrams

- Incidence: Analysis of the 2D interaction diagrams reveals a high incidence of “unfavorable bumps” (steric clashes) across the top poses.
- Frequently clashing residues: Steric stress is repeatedly observed at TYR 652 (chains A/B/C/D) and SER 624, with additional clashes noted at ALA 653 and PHE 656.
- Link to Metrics: Typically, bumps suggest poor fit. However, in the case of quetiapine, the combination of high Interface Area and frequent bumps suggests a mechanism of “forced occlusion.” The molecule is bulky enough to plug the pore, creating steric stress (clashes) against the channel walls while effectively blocking ion passage due to its sheer size.

##### Norquetiapine – Unfavorable-bump readout from 2D diagrams

- Incidence: Norquetiapine poses exhibit a distinct interaction profile. Bumps are present but less diffuse, and the nature of engagement differs due to the introduction of specific electrostatic contacts (cation-π and π-sulfur).
- Frequently clashing residues: Bumps are primarily localized around TYR 652 and SER 624. Visual inspection confirms that the molecule’s smaller size prevents the extensive multi-residue clashing observed with the parent compound.
- Link to Metrics: The lower Area (approx. 417 Å^2^) combined with a cleaner binding profile (fewer extensive clashes) indicates that norquetiapine fits into the binding site without fully occluding the pore. It binds with high affinity (Vina) but lacks the steric bulk to create the “plug” effect seen with quetiapine.

##### Quetiapine & Norquetiapine – Side-by-side comparison centered on steric effects

**Fig. 9:**
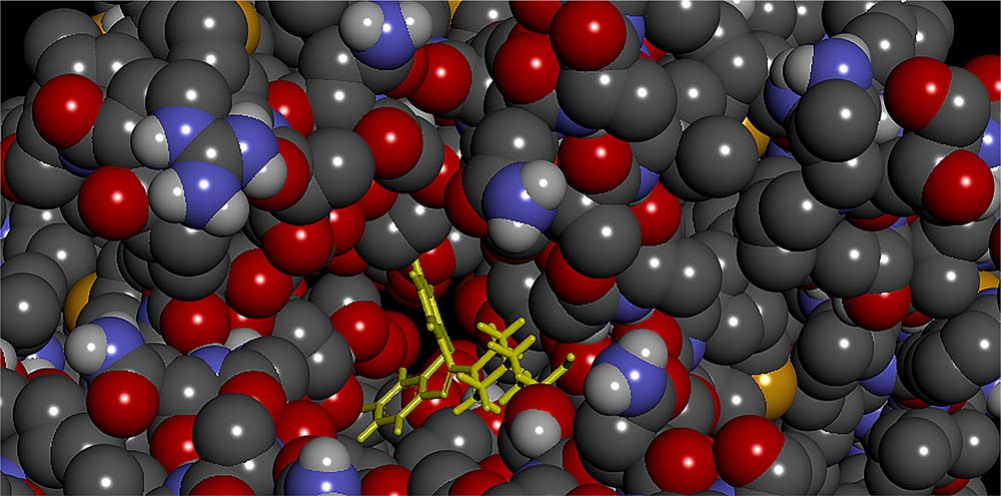
Ball-and-stick structure of quetiapine.

**Fig. 10:**
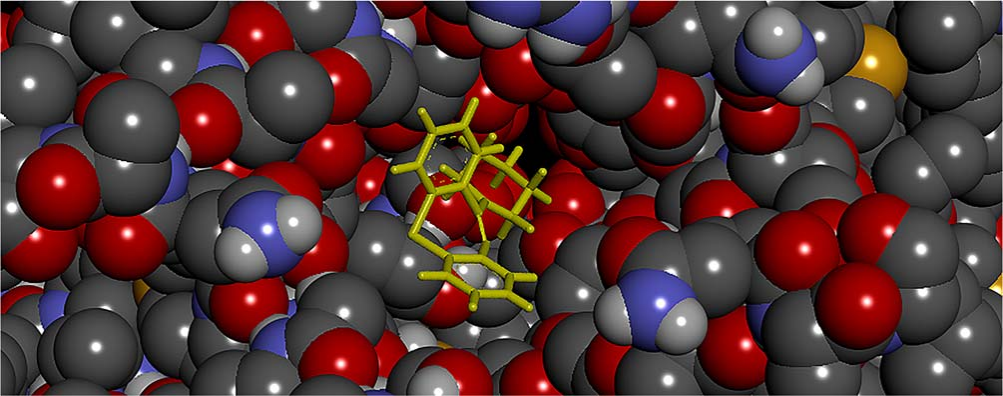
Ball-and-stick structure of norquetiapine.

- The data present a clear distinction in binding modes. Quetiapine acts as a “Steric Blocker”: its high PatchDock score and area demonstrate that it physically fills the pore volume, causing widespread steric clashes (bumps) as it wedges into the channel. In contrast, norquetiapine acts as a “Specific Ligand”: it b inds tightly to critical residues (TYR 652) with fewer steric penalties but does not cover enough surface area to completely physically obstruct the pore.
- It is well-established that inhibitors attach to the drug-binding pocket located in the pore region of the hERG channel, thereby blocking ion conduction.^28^ The PatchDock results provide a structural rationale for the differential safety profiles of the two compounds. Consequently, due to its smaller molecular size relative to the parent molecule, norquetiapine does not encompass the pore region to the same extent as quetiapine. This distinction is quantitatively supported by the significantly lower geometric scores (3,812 vs. 4,554) and contact areas (417.5 Å^2^ vs. 576.3 Å^2^) observed for the metabolite. Structurally, this implies that norquetiapine does not physically obstruct the ion conduction pathway as effectively as quetiapine. This difference can be clearly appreciated through the ball-and-stick models of the top-ranking solutions (Fig. 9 and 10), where quetiapine demonstrates a more voluminous occupancy of the cavity.

### Terfenadine and fexofenadine

#### Results of the first docking process

##### Results from AutoDock Vina

AutoDock Vina indicated a modest affinity advantage for fexofenadine over terfenadine in the top-scoring mode (Mode 1): −9.3 vs. −8.9 kcal/mol, respectively (Table 9). Complete AutoDock Vina outputs for both ligands are found in Supplementary Figs. S12 and S13.

**Table 9:** Binding scores of terfenadine and fexofenadine.

Parent / Metabolite	Affinity (kcal/mol)
Terfenadine	−8.9
Fexofenadine	−9.3

##### Results from BIOVIA discovery studio visualizer

**Fig. 11:**
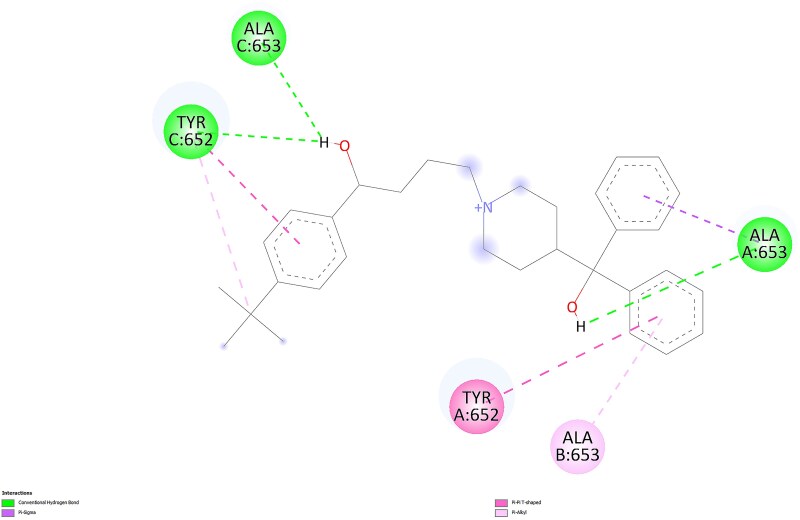
2D interactions of terfenadine with the protein in the most favorable conformation.

**Fig. 12:**
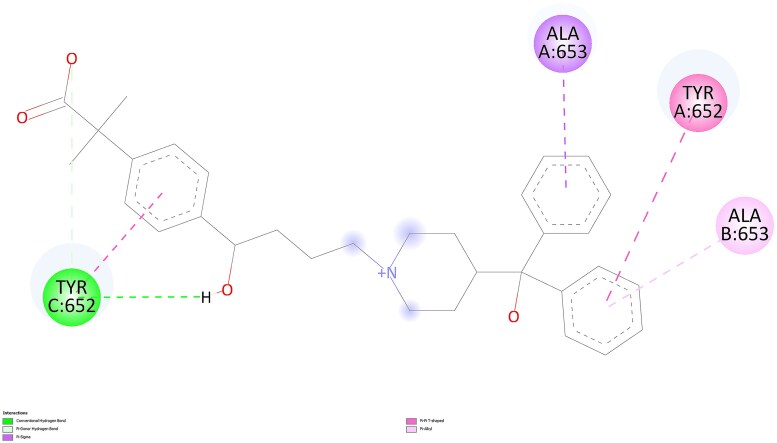
2D interactions of fexofenadine with the protein in the most favorable conformation.

For the most favorable conformations, ligand-protein interactions for terfenadine and fexofenadine are presented in 2D in Figs. 11 and 12, respectively, as visualized using BIOVIA Discovery Studio Visualizer.

Terfenadine engages the hERG pocket with mixed polar–aromatic contacts in the TYR 652 region. Conventional hydrogen bonds are observed from its terminal alcohol groups toward TYR 652, alongside π contacts (π–T shaped/π–alkyl) from aromatic rings to TYR 652 and nearby hydrophobic residues. This pattern is consistent with the manuscript description that both ligands show π interactions at Y652 while terfenadine exhibits additional contacts in that pocket.

In contrast, fexofenadine preserves similar aromatic engagement around TYR 652 and forms a conventional hydrogen bond to TYR 652; its contacts to ALA 653 are π-type (e.g. π σ/π alkyl) rather than conventional hydrogen bonds. The presence of the TYR 652 hydrogen bond in the metabolite’s pose, together with comparable aromatic interactions, provides a plausible structural rationale for its slightly stronger Vina score and suggests improved geometric compatibility within the pocket. Taken together, the Mode 1 visuals indicate that fexofenadine retains the aromatic network seen for terfenadine while presenting a more mixed polar–aromatic profile focused around TYR 652 (with π contacts at ALA 653), which may contribute to the observed affinity difference. However, despite this higher Vina score, fexofenadine is known not to inhibit hERG, whereas terfenadine does. The available data indicate that additional factors such as pharmacokinetics, membrane permeability, and channel accessibility must be considered to explain this discrepancy. To investigate the structural component of this phenomenon and contextualize the energy-based findings, we incorporated a second docking strategy focused on shape complementarity and steric effects.

#### Results of the second docking process

##### Results from PatchDock and BIOVIA discovery studio visualizer

To complement the affinity-based findings from AutoDock Vina, a geometric docking study was performed using PatchDock to evaluate shape complementarity, contact surface area, and steric compatibility within the hERG binding pocket. The top 10 solutions of terfenadine and fexofenadine with PatchDock metrics are shown in Tables 10 and 11 respectively. Complete PatchDock results regarding top 10 solutions including the type of interactions for both ligands (2D diagrams) are found in Supplementary Figs. S14 and S15.

**Table 10:** PatchDock metrics of terfenadine with score, area, and ACE.

Solution number	Score	Area	ACE
1	5,406	698.1	−296.81
2	5,180	629.6	−250.94
3	5,066	600.9	−223.38
4	4,722	604.0	−265.22
5	4,682	542.0	−196.89
6	4,658	563.5	−187.68
7	4,654	503.7	−217.59
8	4,650	544.2	−240.29
9	4,558	519.3	−192.27

**Table 11:** PatchDock metrics of fexofenadine with score, area, and ACE.

Solution number	Score	Area	ACE
1	5,718	680.3	−305.2
2	5,538	694.5	−277.66
3	5,124	556.6	−181.75
4	5,014	584.9	−237.15
5	4,982	622.7	−267.3
6	4,858	583.4	−256.93
7	4,850	670.3	−285.0
8	4,806	530.6	−231.54
9	4,790	642.1	−290.52
10	4,758	760.8	−423.02

##### Interpretation of PatchDock metrics

- Score & Area: Both compounds show high geometric scores and large contact areas. Terfenadine achieves a top score of 5,406 with an area of 698.1 Å^2^. Fexofenadine, however, reaches even higher scores (5718) and comparable areas, indicating that both molecules are large enough to occupy the pore region extensively.
- ACE: A crucial difference appears in the ACE values. Terfenadine’s solutions generally show shallower ACE values (many in the −180 to −250 range). In contrast, fexofenadine achieves significantly deeper negative ACE values in several solutions, reaching as low as −423.02 (Solution 10) and − 305.2 (Solution 1). This suggests that while both occupy the space, fexofenadine can find a much more energetically favorable atomic packing than the parent drug.

##### Terfenadine – Unfavorable-bump readout from 2D diagrams

- Incidence: Terfenadine displays a “High Incidence” of steric clashes. Visual analysis reveals that 9 out of the top 10 solutions exhibit unfavorable bumps (red dashed lines). Only Solution 9 appears relatively clean.
- Frequently clashing residues: The steric stress is severe and targets the most critical residues for hERG blockage. Widespread clashes are observed against TYR 652 (seen in Solutions 2, 3, 4) and SER 660 / SER 624 (seen in Solutions 1, 5, 7, 8). This indicates a “Forced Fit” mechanism.
- Link to Metrics: The combination of high Contact Area (~698 Å^2^) but poor/shallow ACE values correlates perfectly with the visual evidence of bumps. Terfenadine effectively “plugs” the pore due to its volume, but it does so under significant steric conflict, wedging itself against the channel walls (TYR652) rather than fitting harmoniously.

##### Fexofenadine – Unfavorable-bump readout from 2D diagrams

- Incidence: Fexofenadine presents a more complex but distinct profile. While bumps are present in some poses (due to the molecule’s large size), there are notable solutions that are remarkably “Clean” and free of steric conflicts, such as Solution 3 and Solution 6.
- Frequently clashing residues: When bumps do occur (e.g. Solution 10), they are localized, but the molecule demonstrates the ability to adopt conformations (like Solution 6) that engage the pocket without the diffuse steric stress seen in the parent.
- Link to Metrics: The existence of these “clean” poses aligns with the deeply negative ACE values (e.g. −305, −423). Unlike Terfenadine, which must clash to occupy the pore, Fexofenadine is geometrically capable of a “Relaxed Fit.”

##### Terfenadine & Fexofenadine – Side-by-side comparison

The structural analysis resolves the paradox of Fexofenadine’s high affinity scores versus its clinical safety:

- Terfenadine (The Steric Blocker): Acts through “Forced Occlusion.” It creates a massive physical block (High Area) but generates widespread steric stress (Frequent Bumps against TYR652/SER660). It wedges into the pore like a mismatched plug, physically obstructing the ion pathway through steric bulk.
- Fexofenadine (The Relaxed Occupant): Acts through “Geometric Compatibility.” Its deep ACE values and the presence of bump-free conformations suggest it fits the pocket with high stability. However, this “snug” fit, likely stabilized by its zwitterionic nature (carboxylate group), avoids the specific “wedging” stress that characterizes the parent’s blockage.
- Consequently, Terfenadine’s profile of High Area + High Steric Clashes defines its role as a potent hERG blocker, whereas Fexofenadine’s ability to bind with Deep ACE + Relaxed Geometry allows it to interact with the protein without causing the functional occlusion or allosteric disruption typical of the parent drug.

##### The “Zwitterionic Safety Switch”: Why fexofenadine is safer despite high affinity

Our geometric docking results support this distinction. Terfenadine docking poses showed frequent clashes deep within the pocket (TYR652, PHE656), consistent with a molecule forcing its way into a hydrophobic environment. Fexofenadine, containing the polar carboxylate tail, seeks a more “relaxed” geometry. Even when it binds in silico (hence the high score), its polar tail likely prevents the deep, destabilizing hydrophobic burial that terfenadine achieves. The “Clean” poses observed in PatchDock suggest that even if it interacts, it does not lock the channel in a stressed, non-conducting state as aggressively as the parent.

As a result, the paradox of “High Affinity but High Safety” is resolved by the zwitterionic brake. While computational docking confirms fexofenadine can fit the pocket with excellent shape complementarity (Deep ACE), its physical inability to cross the membrane efficiently prevents it from reaching the critical concentration needed to block the channel in vivo. Terfenadine lacks this “brake,” leading to the severe steric conflict and hERG blockade identified in our study.

## Discussion

### Integration of geometric and energetic profiling in TdP risk assessment

The most distinguishing aspect of this study is the implementation of a dual-strategy docking approach that synergizes binding affinity estimation (AutoDock Vina) with geometric shape complementarity analysis (PatchDock). Traditional in silico assessments often rely predominantly on binding energy scores. However, our results demonstrate that relying on affinity alone can lead to misleading conclusions when dissociated from the geometric and physicochemical context. By incorporating PatchDock to evaluate steric stress (unfavorable bumps) and atomic contact energy (ACE), we provide a higher-resolution prediction model that distinguishes between “stable binding” and “functional blockade.” This integrated methodology offers a superior vantage point for interpreting TdP risk compared to unidimensional methods.

### Mechanistic insights into Metabolite-Channel interactions

Our multifaceted analysis reveals that the risk of TdP is not solely dictated by how tightly a molecule binds, but by how it occupies the pore and its physicochemical access to the site.

- Steric Blockade vs. Specific Binding (Quetiapine Case): The comparison between quetiapine and norquetiapine illustrates the importance of steric analysis. The parent drug, quetiapine, acts as a “Steric Blocker” Its high geometric score (4554) and extensive contact area (up to 588.8 Å^2^) correlate with a high incidence of steric bumps against critical pore residues TYR652 and SER624. This suggests a mechanism of forced occlusion. In contrast, the metabolite norquetiapine functions as a “Specific Ligand” despite a comparable Vina affinity (−7.7 kcal/mol), it exhibits a significantly smaller interface area (~417 Å^2^) and a cleaner binding profile. This lack of steric bulk implies that the metabolite does not physically occlude the ion pathway as effectively as the parent, aligning with literature suggesting its distinct electrophysiological profile.
- The Affinity Paradox and Physicochemical Barriers (Terfenadine Case): A critical finding of this study is that high binding affinity does not essentially equate to high in vivo toxicity if the molecule is kinetically restricted. Fexofenadine presented a higher binding affinity (−9.3 kcal/mol) compared to its cardiotoxic parent terfenadine (−8.9 kcal/mol), along with deep ACE values (−423.02) indicating excellent shape complementarity. Paradoxically, it is clinically safe. This discrepancy is resolved by the “Zwitterionic Safety Switch.” Unlike the highly lipophilic terfenadine, which penetrates the membrane to cause “forced occlusion” (evidenced by widespread steric clashes), fexofenadine possesses a carboxylate group that renders it zwitterionic. This physicochemical property creates a barrier to membrane permeability, effectively preventing the “geometric compatibilist” from accessing the intracellular binding site in sufficient concentrations.
- Metabolite-Driven Potency (Astemizole Case): In contrast to fexofenadine, the metabolism of astemizole to desmethylastemizole represents a “toxicity maintenance” scenario. The metabolite demonstrated a superior binding affinity (−9.2 kcal/mol) compared to the parent (−8.8 kcal/mol). Crucially, PatchDock analysis revealed that desmethylastemizole achieves this high affinity with a “relaxed” geometry (Deep ACE: −338.36) and significantly fewer steric bumps. This indicates that the metabolite stabilizes the block more effectively than the parent drug, providing a structural rationale for why desmethylastemizole contributes significantly to the arrhythmogenic profile.

### Proposed criteria for enhanced TdP risk assessment

Based on the synthesis of our docking parameters and the observed physicochemical determinants, we propose a six-point evaluation criteria for identifying high-risk metabolites in early drug development:

- Affinity Match: Does the metabolite exhibit a binding affinity comparable to or greater than the parent?
- Interaction Potential: Does it show increased specific interactions (e.g. H-bonds, pi-stacking) with key pore residues?
- Complex Stability: Does the metabolite form geometrically stable complexes (indicated by deeper ACE values) compared to the parent?
- Physicochemical Access: Does the metabolite possess properties (e.g. lipophilicity vs. zwitterionic nature) that facilitate or hinder access to the intracellular pore?
- Steric Occlusion: Does the metabolite physically occlude the pore region (High Contact Area) similarly to the parent?
- Geometric Fit: Does it bind with a “relaxed” geometry (low steric clashes), potentially indicating a prolonged blockade?

### Clinical and regulatory implications

The findings of this study underscore that TdP risk assessment cannot be confined to the parent compound alone. The divergent profiles of the three pairs analyzed—where metabolites can be safer (fexofenadine), mechanistically distinct (norquetiapine), or equally potent (desmethylastemizole)—highlight the necessity of a holistic approach. We advocate for the integration of these six criteria into preclinical safety workflows. Such a comprehensive strategy, combining affinity, geometry, and physicochemical properties, will enhance the predictive accuracy of cardiotoxicity screenings, ultimately reducing late-stage attrition and improving patient safety in new drug development.

## Conclusion

The accurate prediction of TdP risk remains a critical challenge in drug safety. This study demonstrates that the traditional focus on hERG inhibition by the parent compound alone is insufficient. By employing an integrated computational framework that combines binding affinity (AutoDock Vina), geometric shape complementarity (PatchDock), and physicochemical profiling (ADMET), we have shown that active metabolites can significantly alter the cardiotoxicity profile of a drug—either by maintaining potency (astemizole), mitigating risk through steric differences (quetiapine), or acting as a safety switch through kinetic restriction (terfenadine).

Our findings highlight that “high affinity” does not always equate to “high risk” if the molecule lacks the geometric capacity for forced occlusion or the physicochemical ability to access the target. The “Zwitterionic Safety Switch” identified in fexofenadine serves as a prime example of why ADMET properties must be evaluated alongside binding scores. Furthermore, the geometric distinction between “steric blockers” and “specific ligands” provides a structural rationale for the divergent clinical profiles of drugs and their metabolites.

We conclude that a multi-dimensional approach is essential for modern risk assessment. We strongly recommend that regulatory guidelines and early-stage drug discovery protocols explicitly incorporate the evaluation of metabolites using the six-point criteria proposed in this study. While in vivo validation remains the gold standard, this comprehensive in silico strategy offers a robust, resource-efficient filter to identify high-risk candidates early in the pipeline. Ultimately, shifting from a parent-centric view to a metabolite-inclusive perspective will enhance the safety margins of new therapeutics and prevent the late-stage withdrawal of promising drugs.

## Supplementary Material

Appendix_A_-_Supporting_Information_tfaf186

## Data Availability

No data was used for the research described in the article.
