# Supplementary material for: Advancing Torsades de pointes risk prediction: unveiling the role of drug metabolites through molecular docking
Source: Toxicol Res (Camb). 2026 Jan 29;15(1):tfaf186. doi: 10.1093/toxres/tfaf186 (PMC12853304; doi:10.1093/toxres/tfaf186)
Supplement: Appendix_A_-_Supporting_Information_tfaf186 [file appendix_a_-_supporting_information_tfaf186.docx]

**Advancing Torsades de Pointes Risk Prediction: Unveiling the Role of Drug Metabolites Through Molecular Docking**

**Table of Contents**

[Figure S1.The final prepared protein structure. 4](#_Toc215938731)

[Figure S3.Results of ramachandran plot of the prepared protein. 5](#_Toc215938732)

[Figure S4.Complete AutoDockVina outputs of astemizole. 5](#_Toc215938733)

[Figure S5.Complete AutoDock Vina outputs of desmethlyastemizole. 5](#_Toc215938734)

[Figure S6.Complete PatchDock results regarding the top 10 solutions of astemizole. 8](#_Toc215938735)

[Figure S7.Complete PatchDock results regarding the top 10 solutions of desmethylastemizole. 11](#_Toc215938736)

[Figure S8.Complete AutoDock Vina outputs of quetiapine. 11](#_Toc215938737)

[Figure S9.Complete AutoDock Vina outputs of norquetiapine. 11](#_Toc215938738)

[Figure S10.Complete PatchDock results regarding the top 10 solutions of quetiapine. 14](#_Toc215938739)

[Figure S11.Complete PatchDock results regarding the top 10 solutions of norquetiapine. 17](#_Toc215938740)

[Figure S12.Complete AutoDock Vina outputs of terfenadine. 17](#_Toc215938741)

[Figure S13.Complete AutoDock Vina outputs of fexofenadine. 18](#_Toc215938743)

[Figure S14.Complete PatchDock results regarding the top 10 solutions of terfenadine. 21](#_Toc215938744)

[Figure S15.Complete PatchDock results regarding the top 10 solutions of fexofenadine. 23](#_Toc215938745)


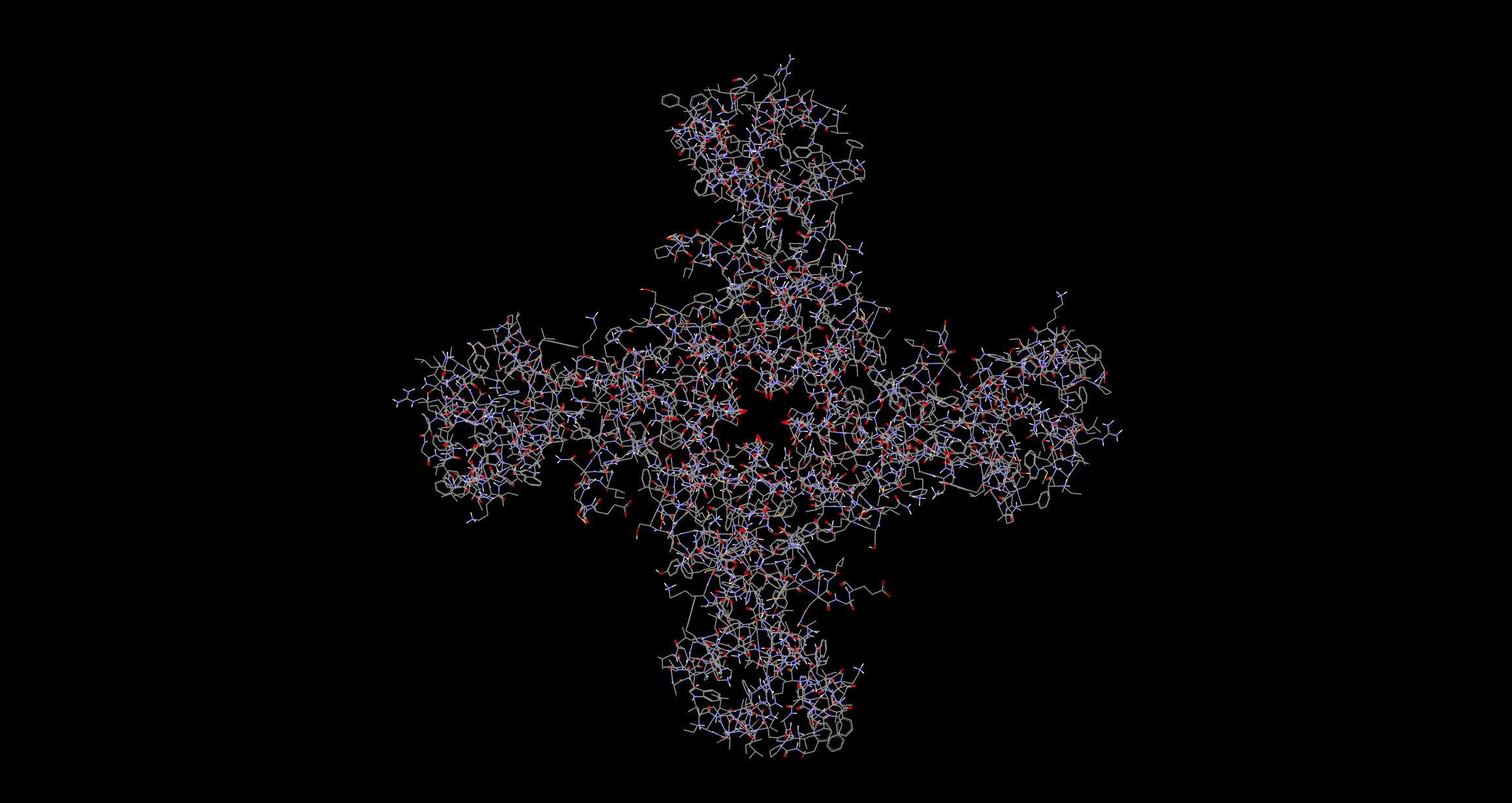


# Figure S1.The final prepared protein structure.


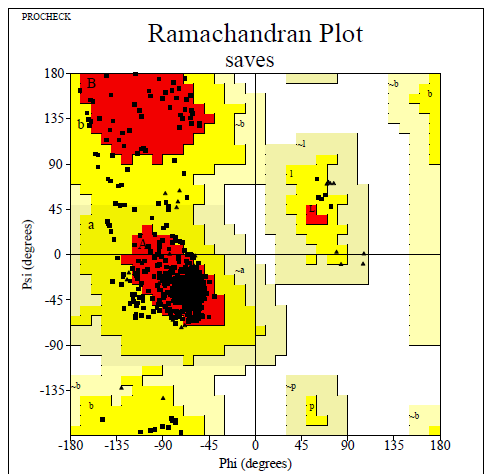


**Figure S2**.The ramachandran plot for the prepared protein.


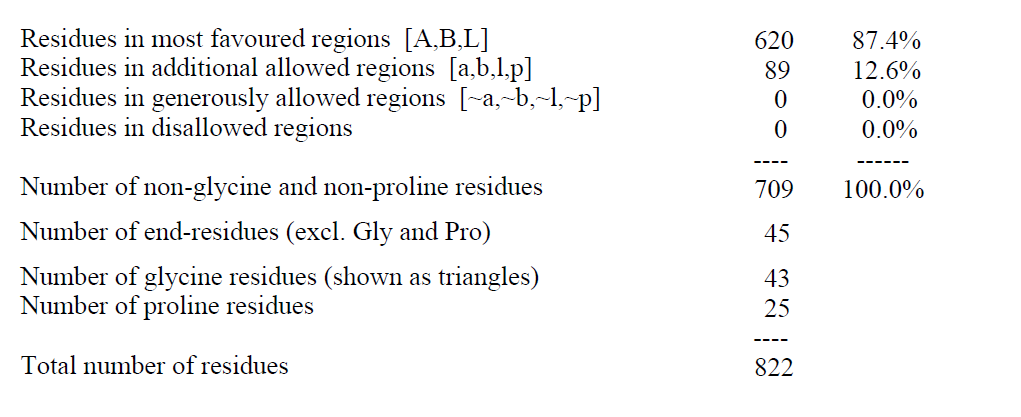


# Figure S3.Results of ramachandran plot of the prepared protein.


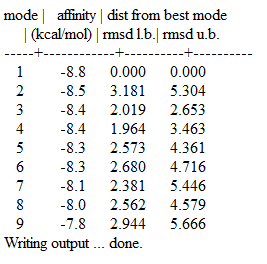


# Figure S4.Complete AutoDockVina outputs of astemizole.


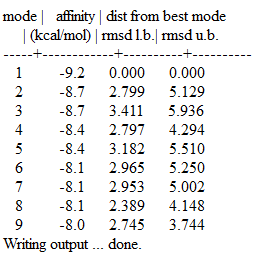


# Figure S5.Complete AutoDock Vina outputs of desmethlyastemizole.


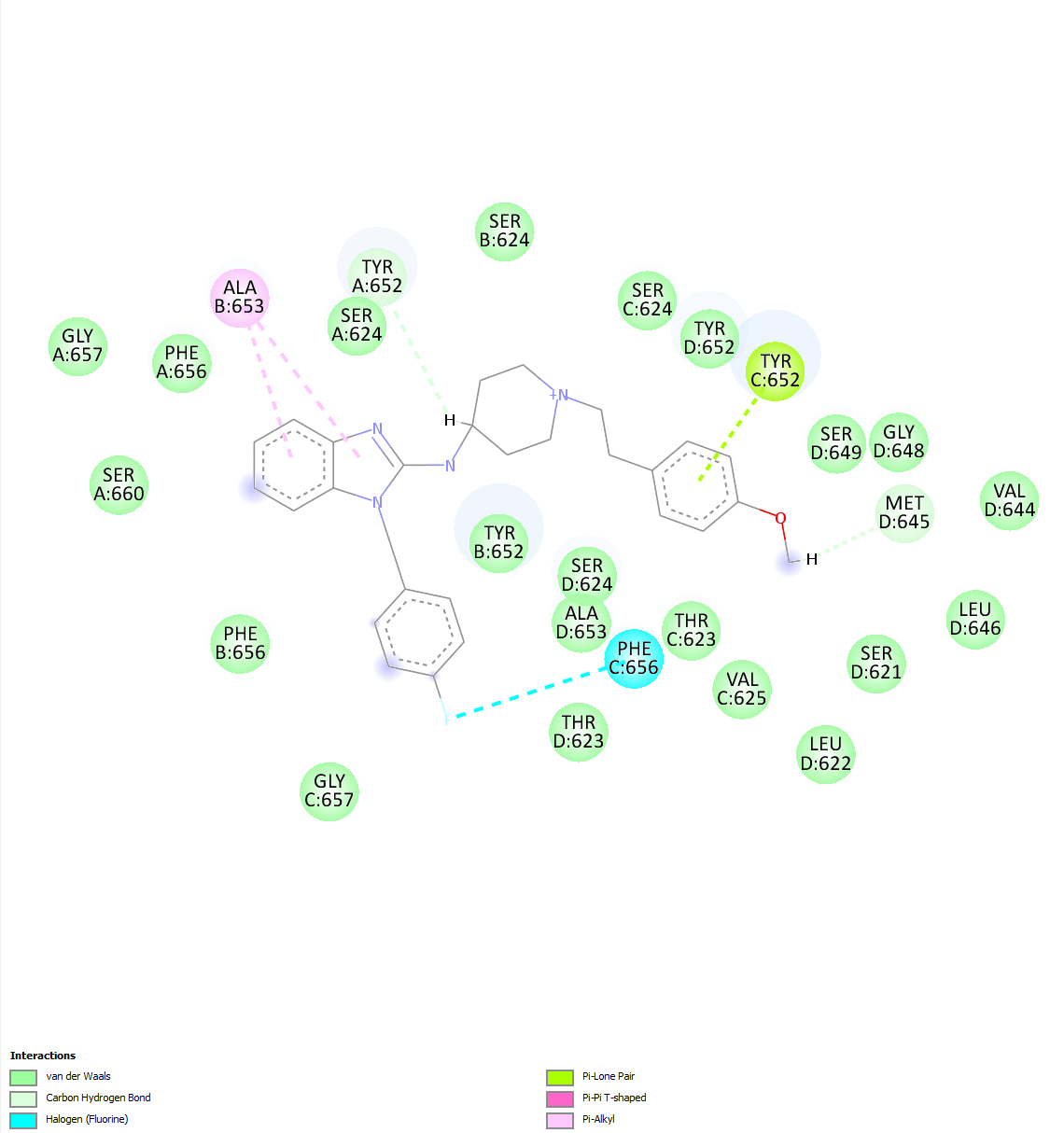

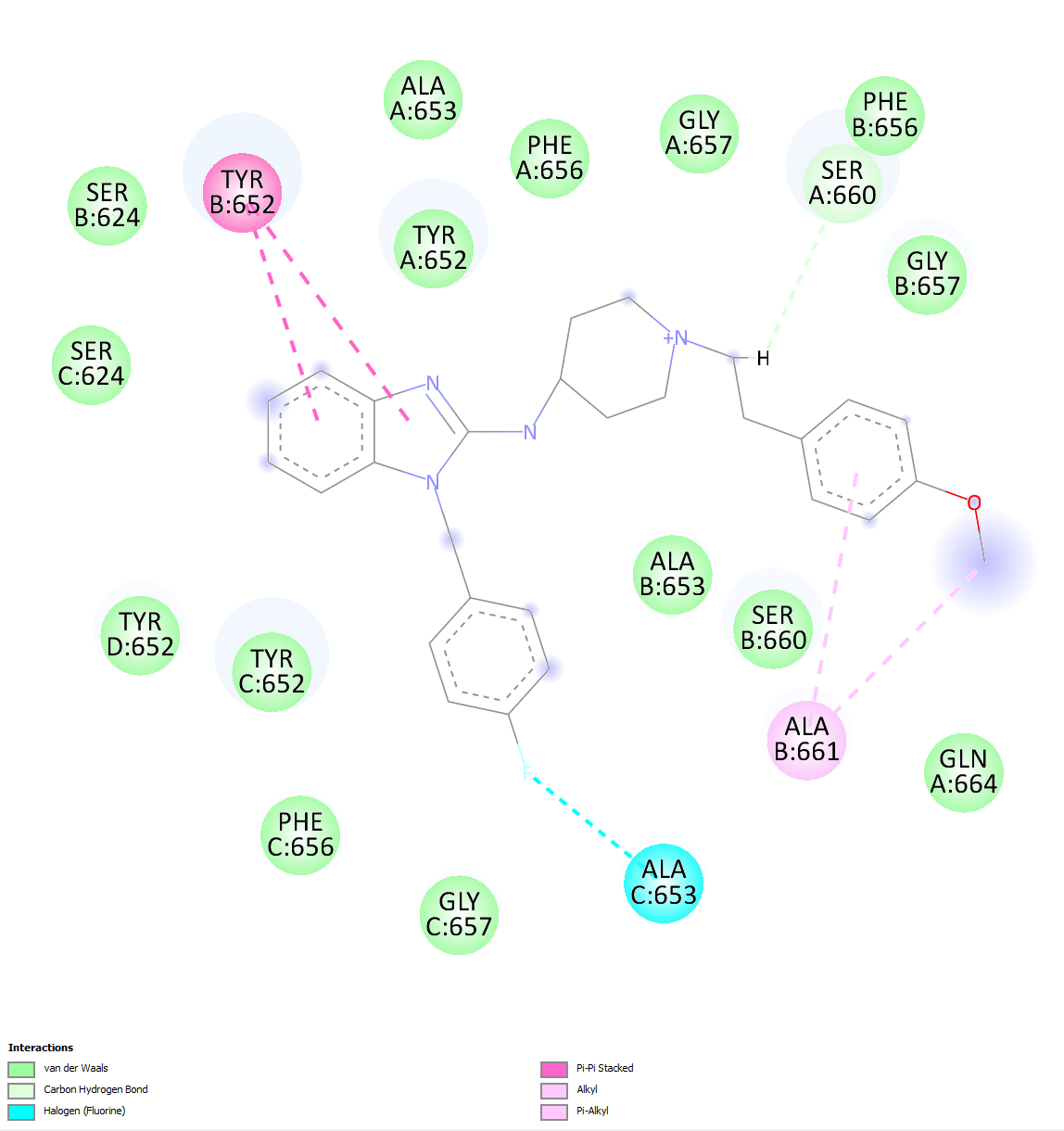


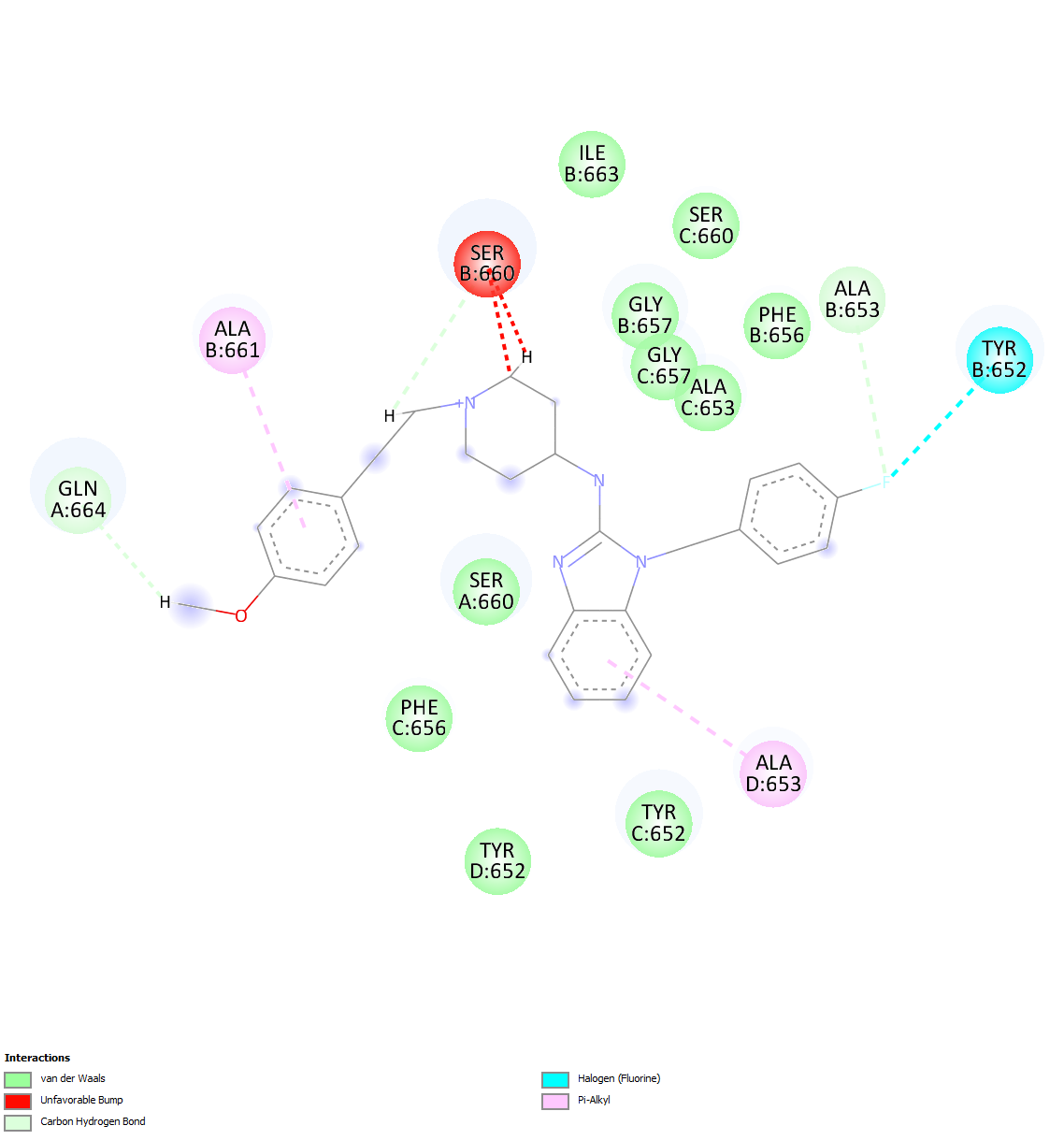

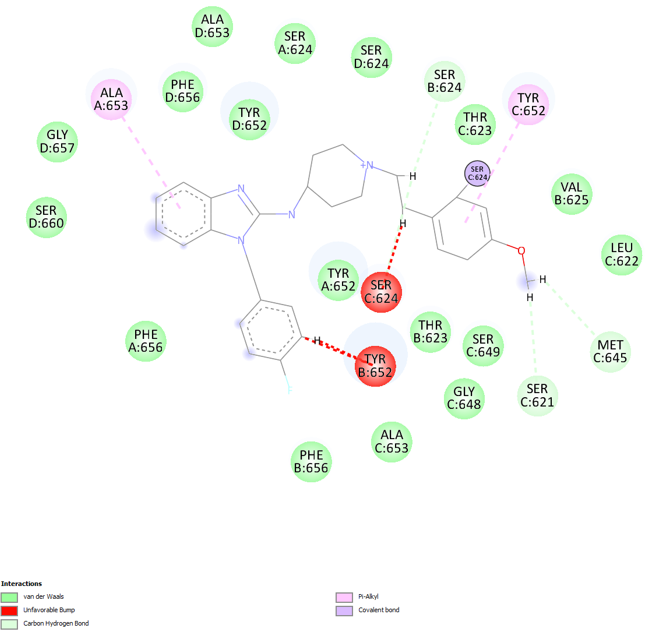


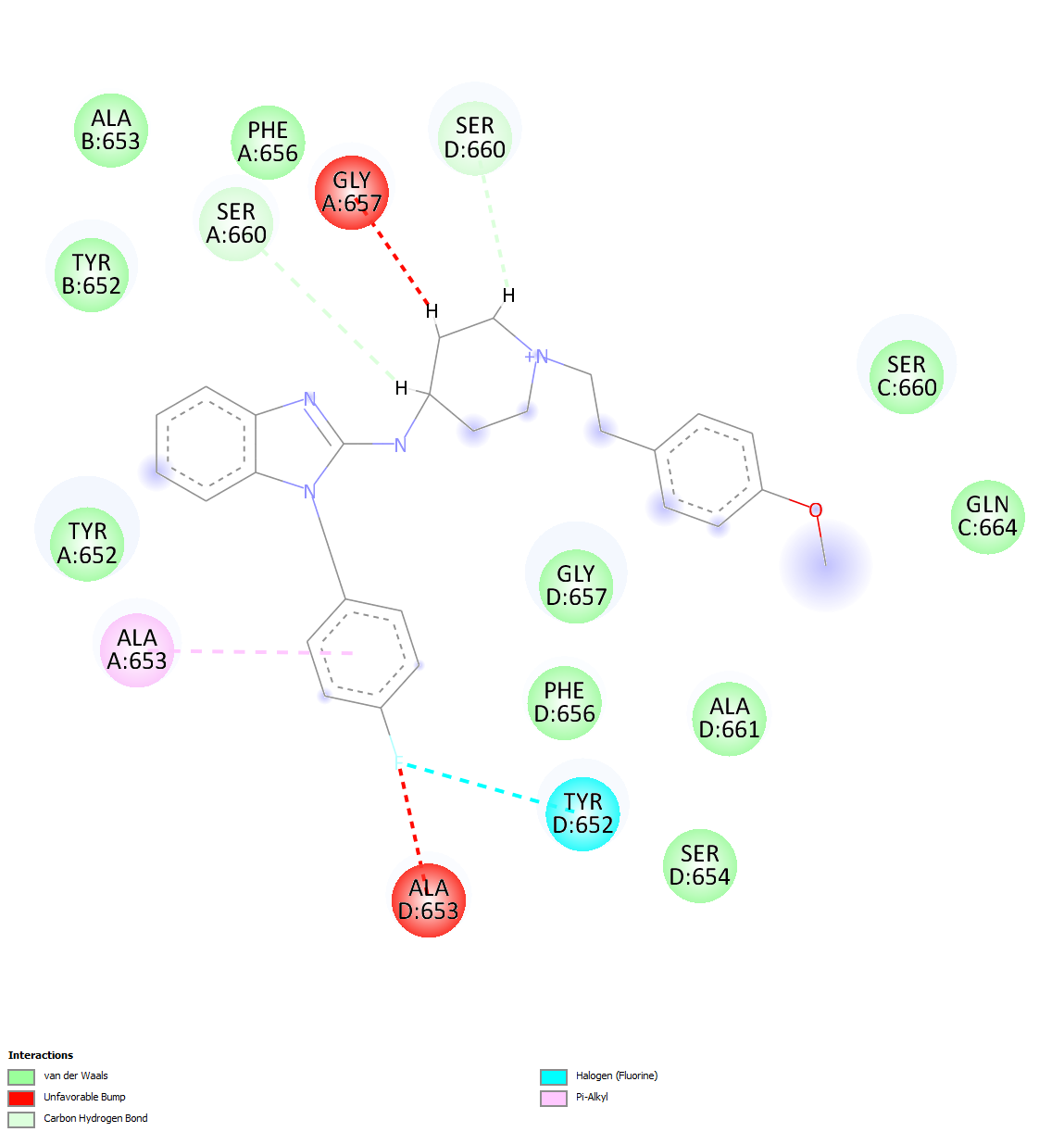

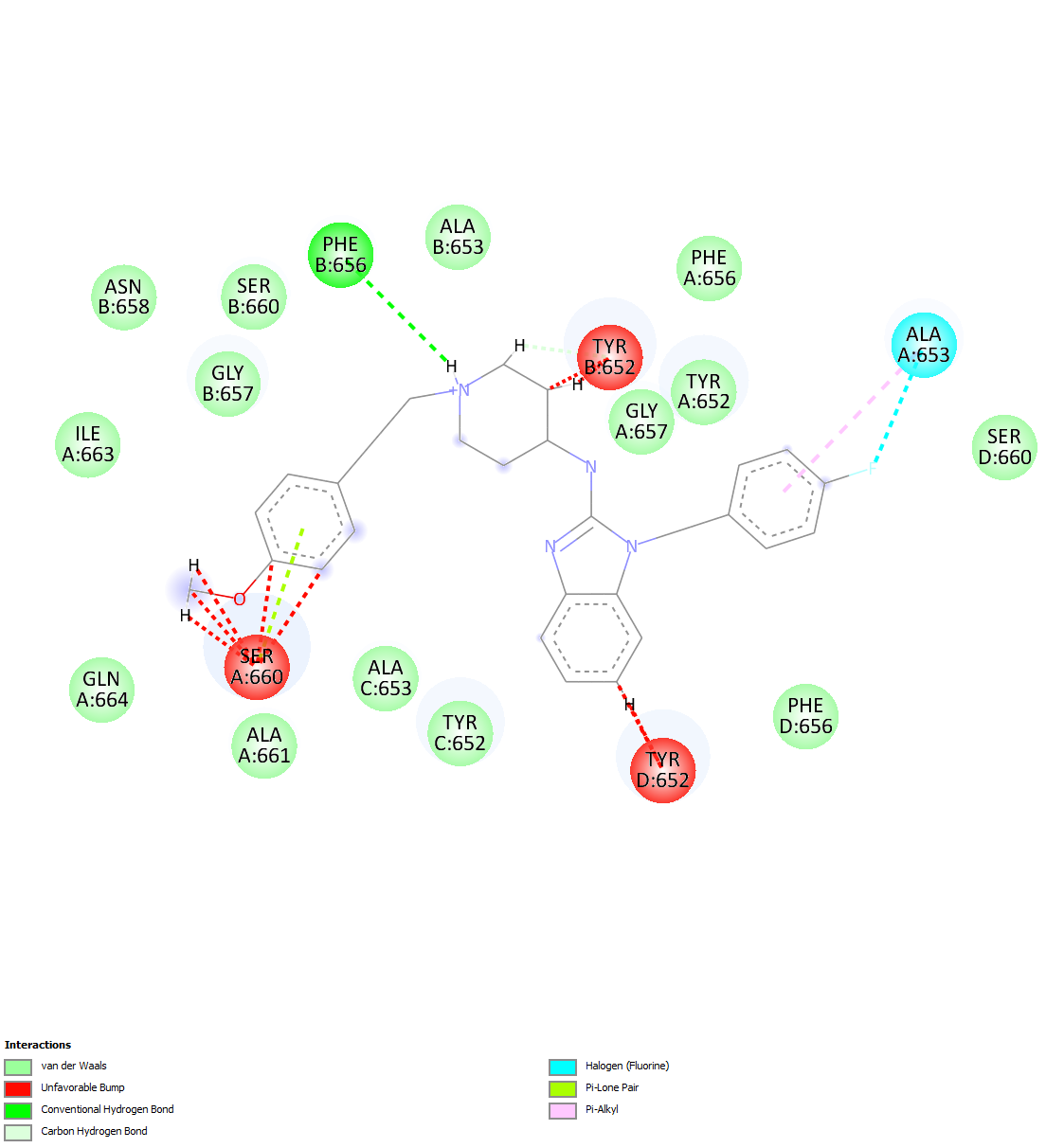


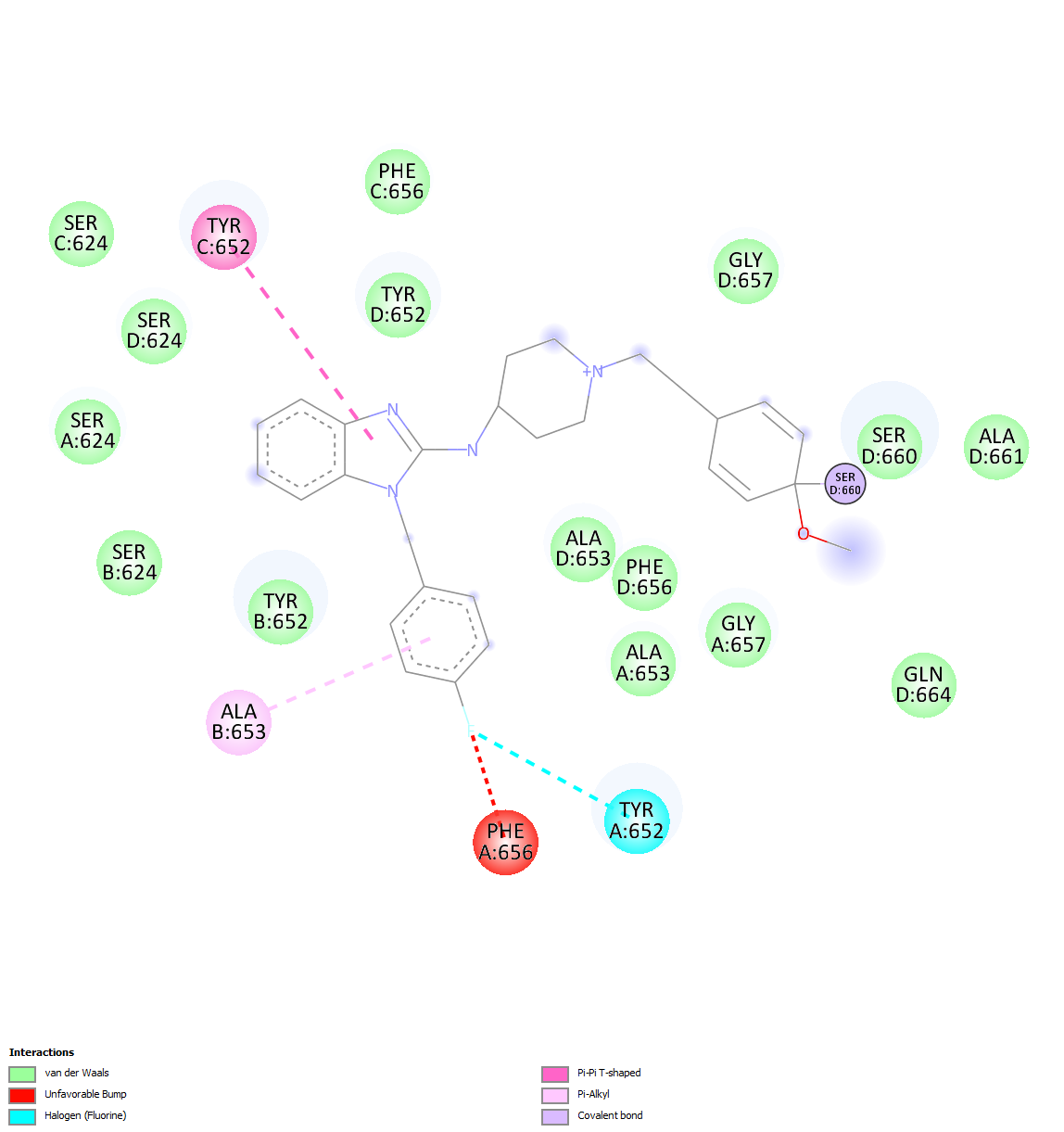

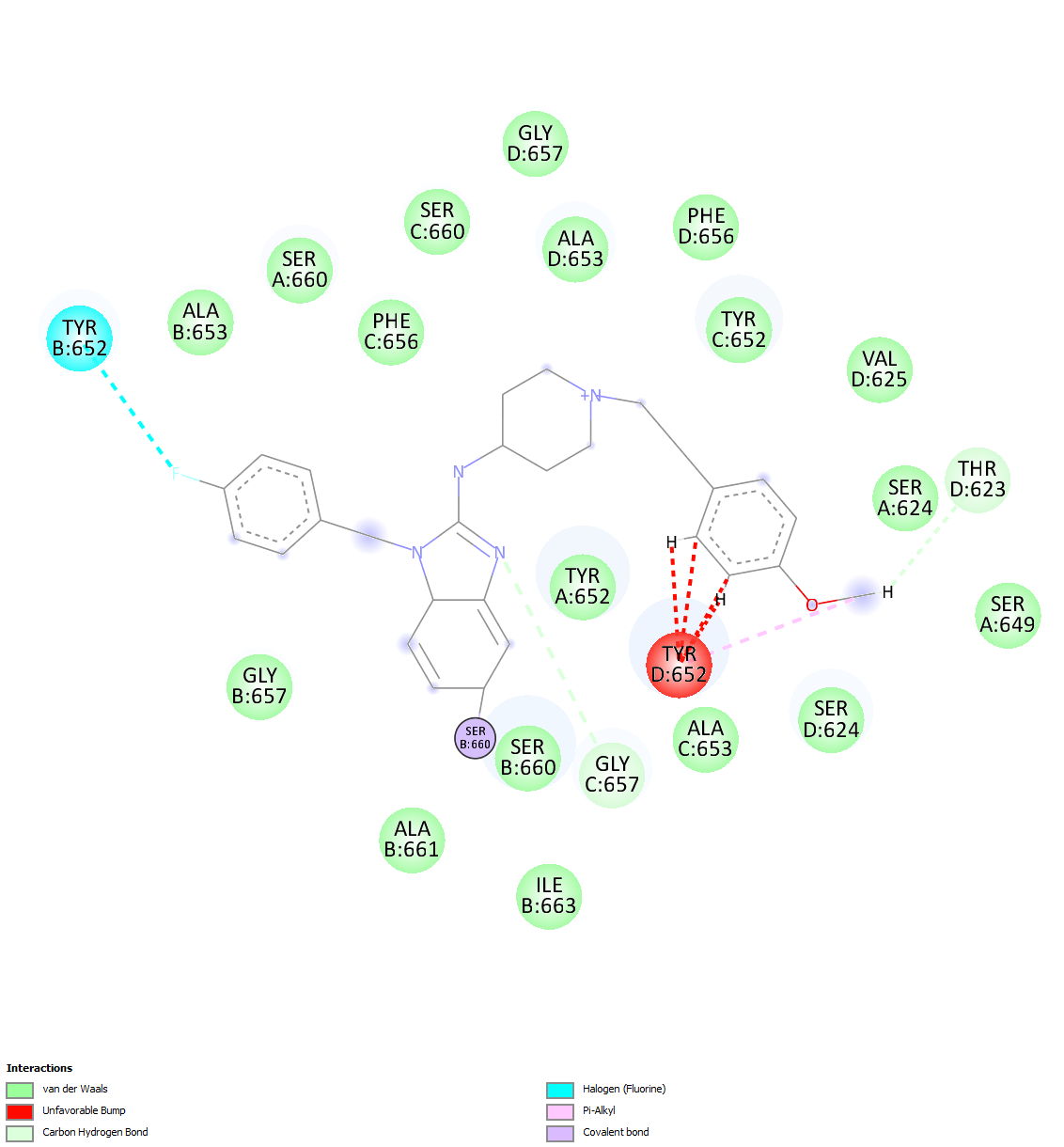


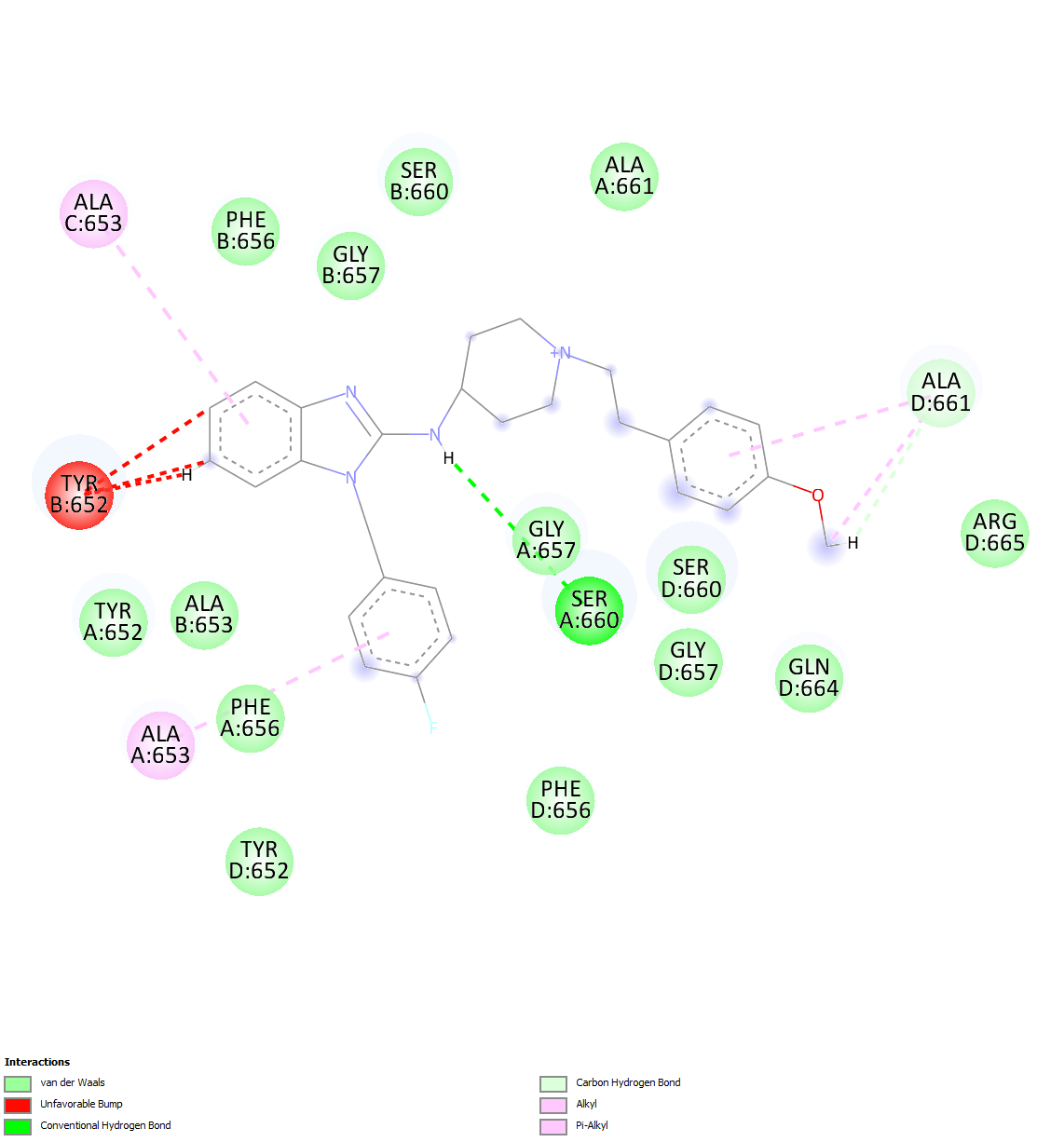

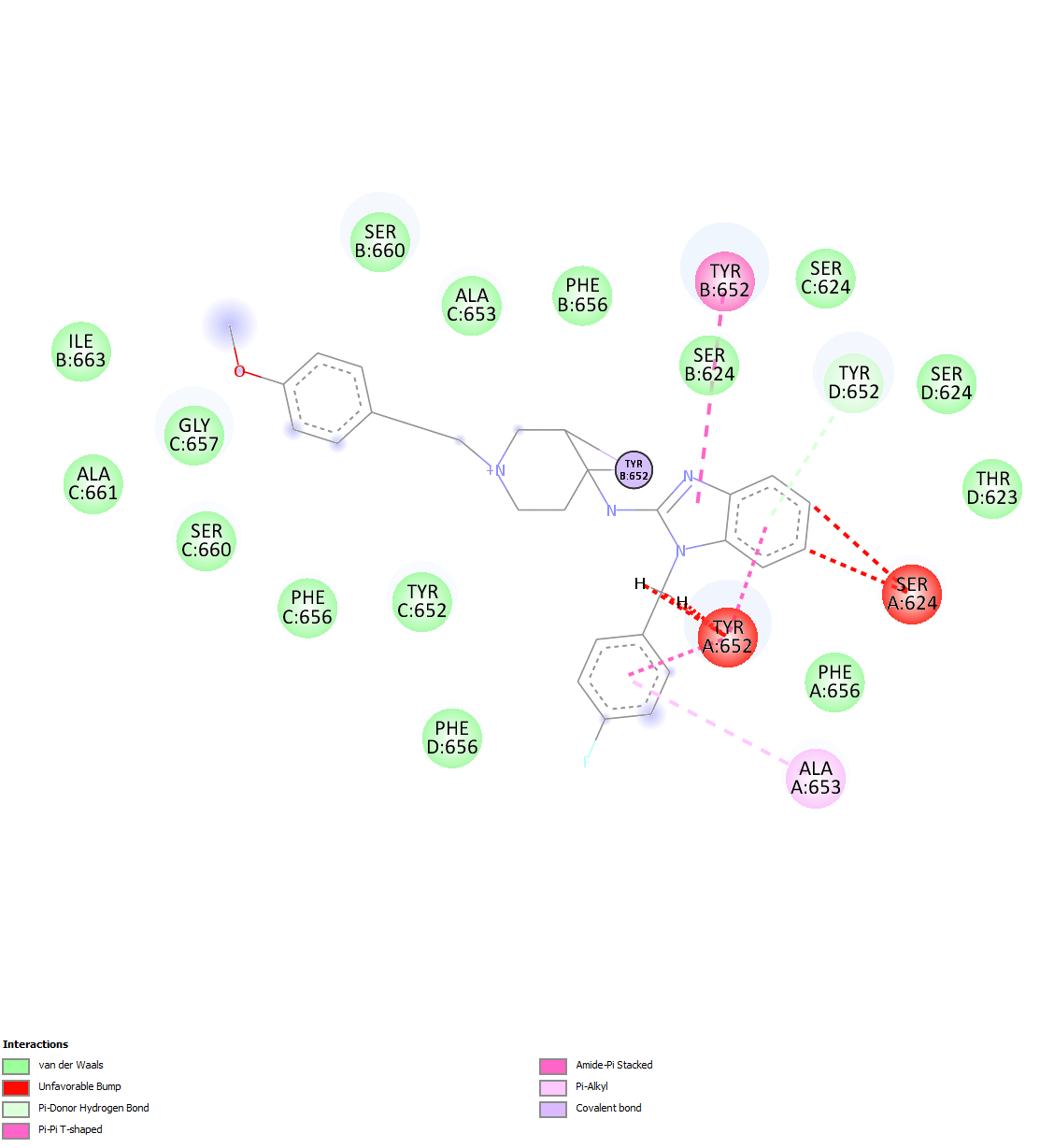


# Figure S6.Complete PatchDock results regarding the top 10 solutions of astemizole.


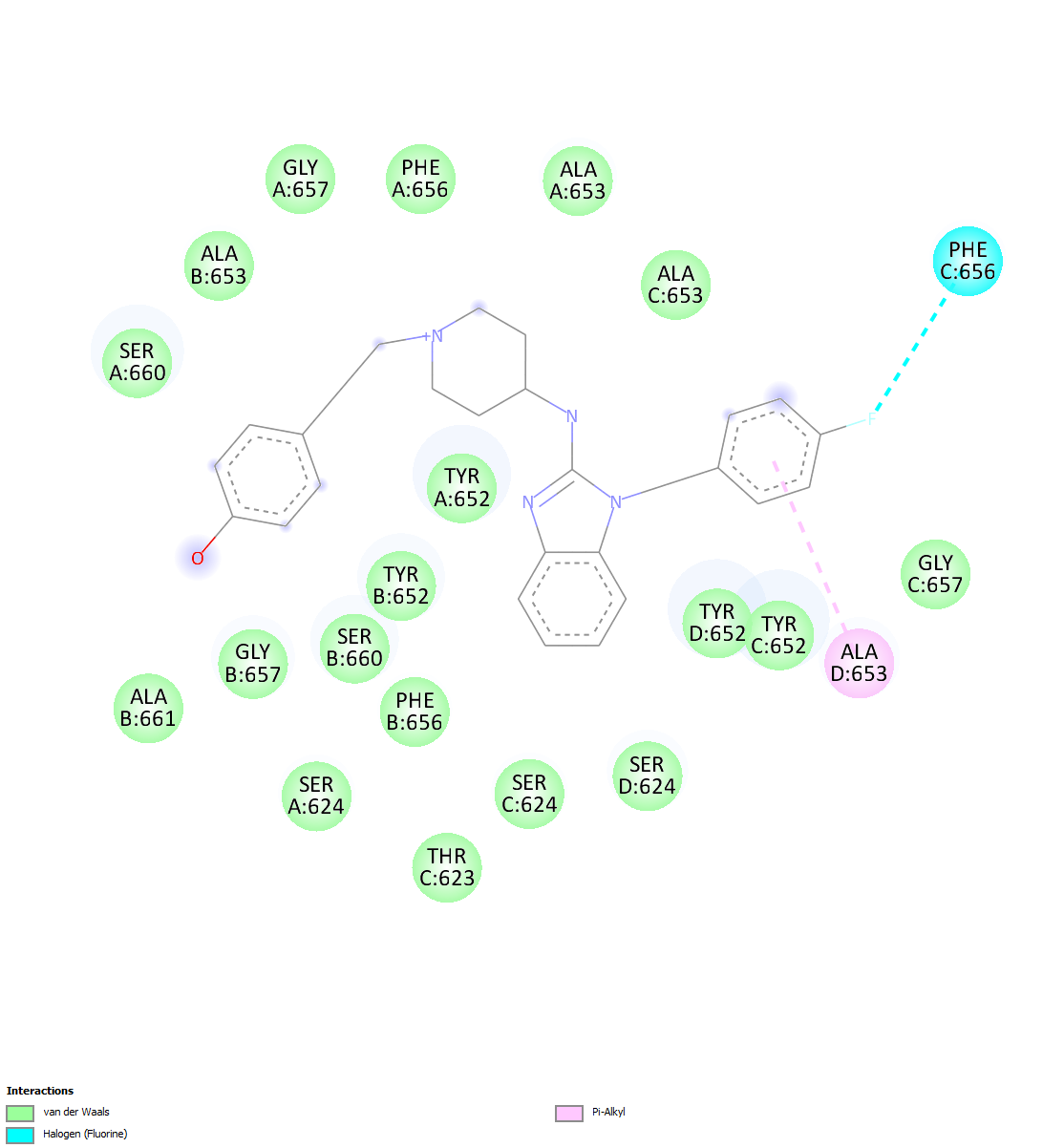

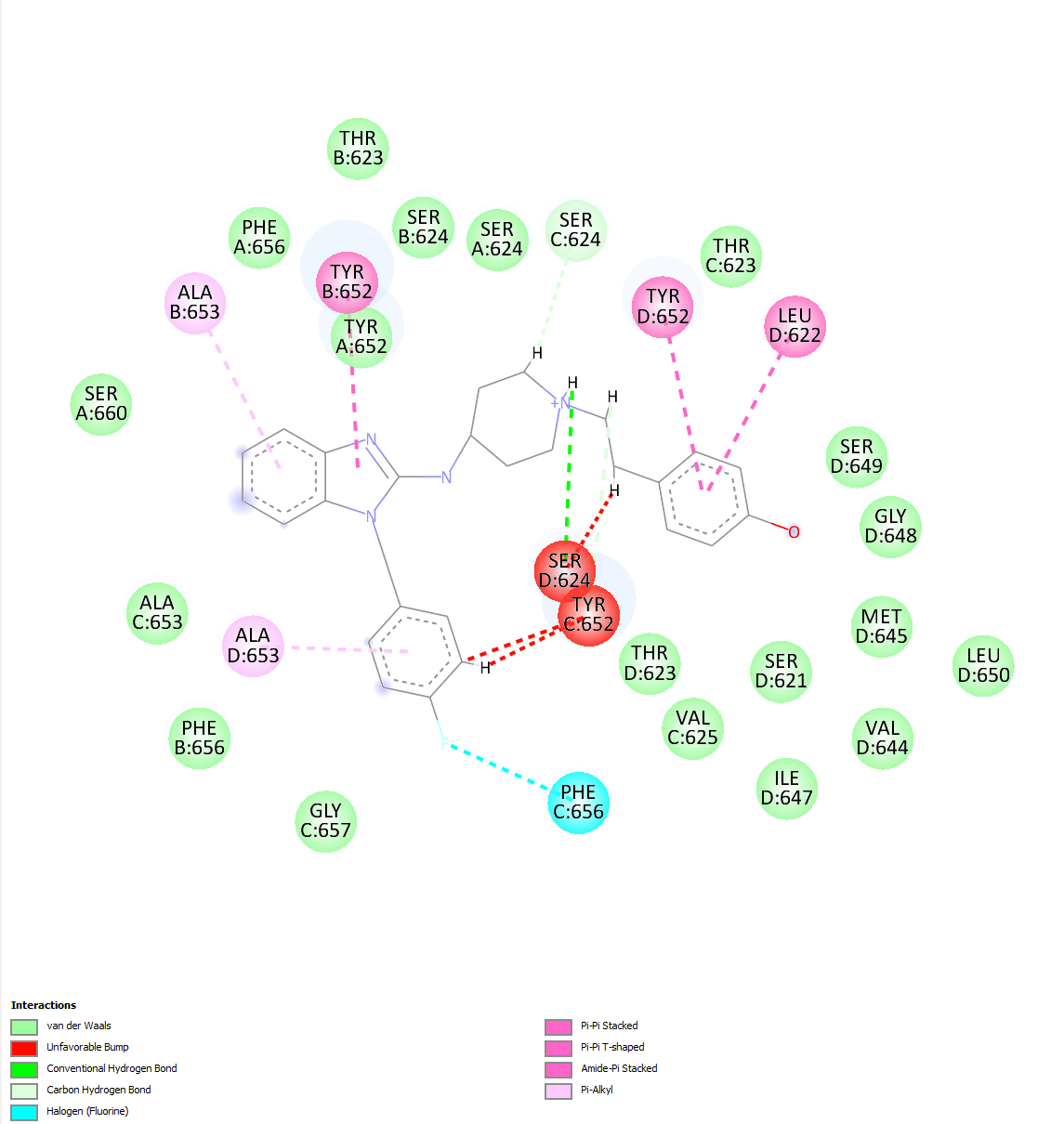


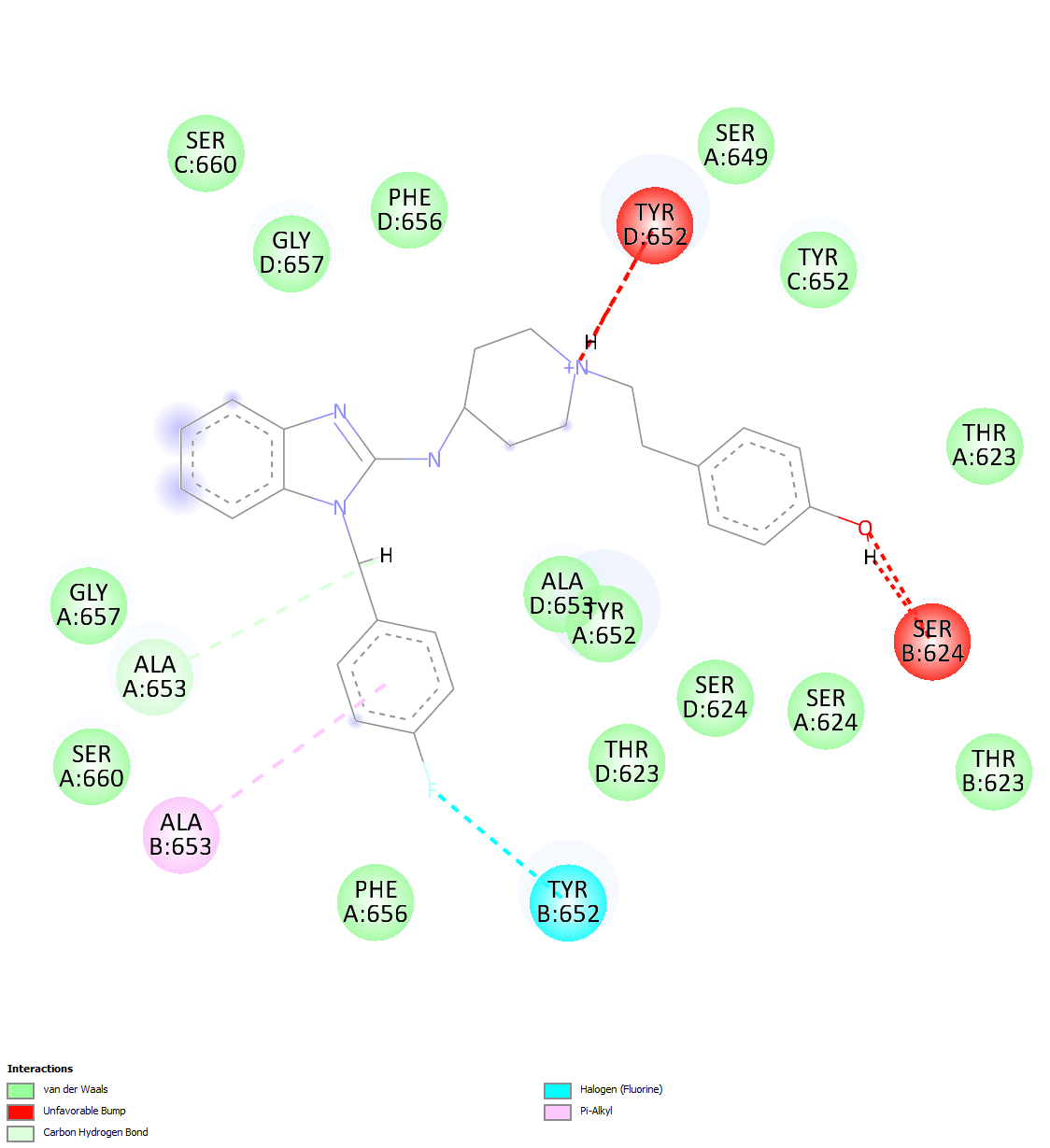

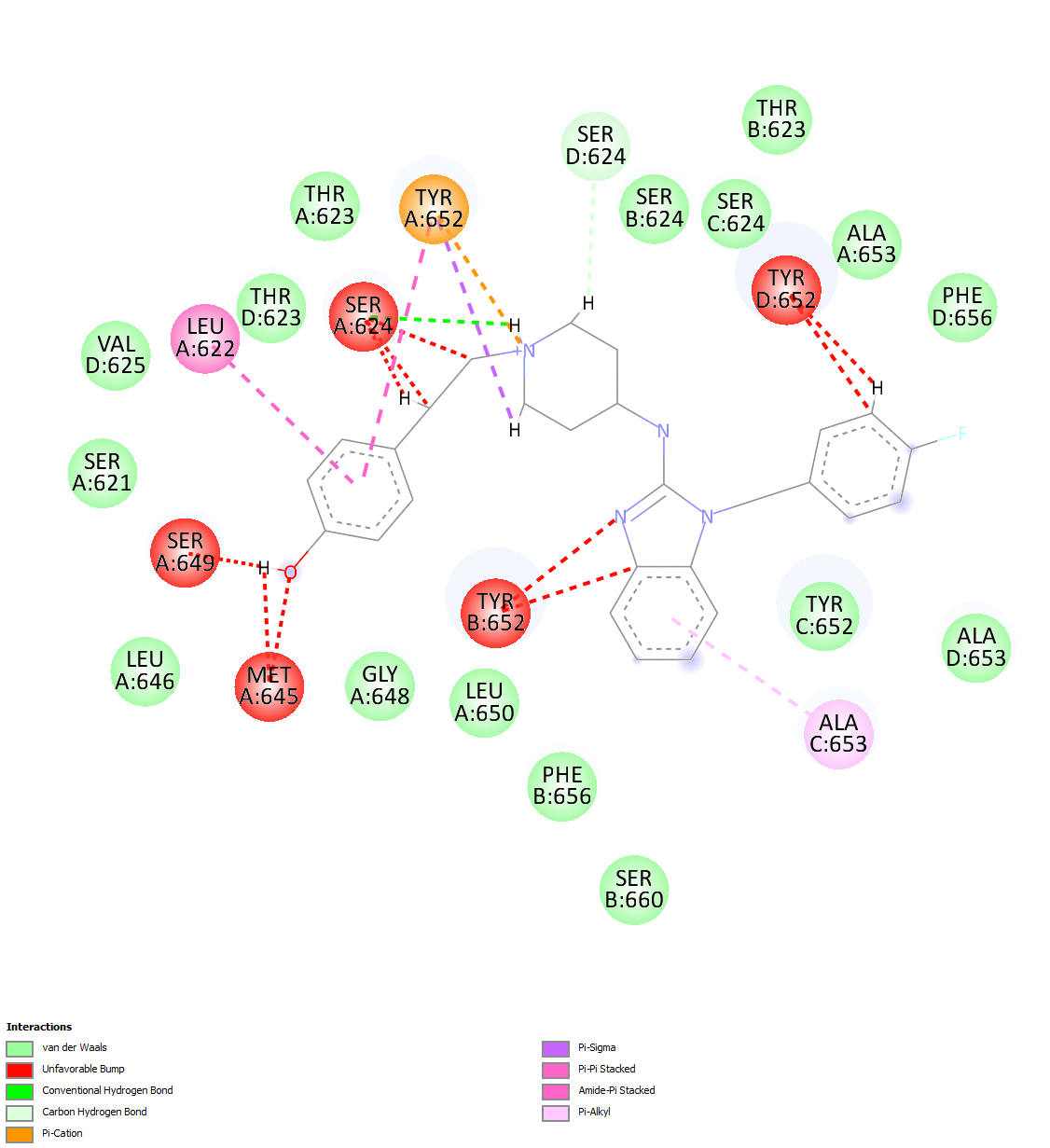


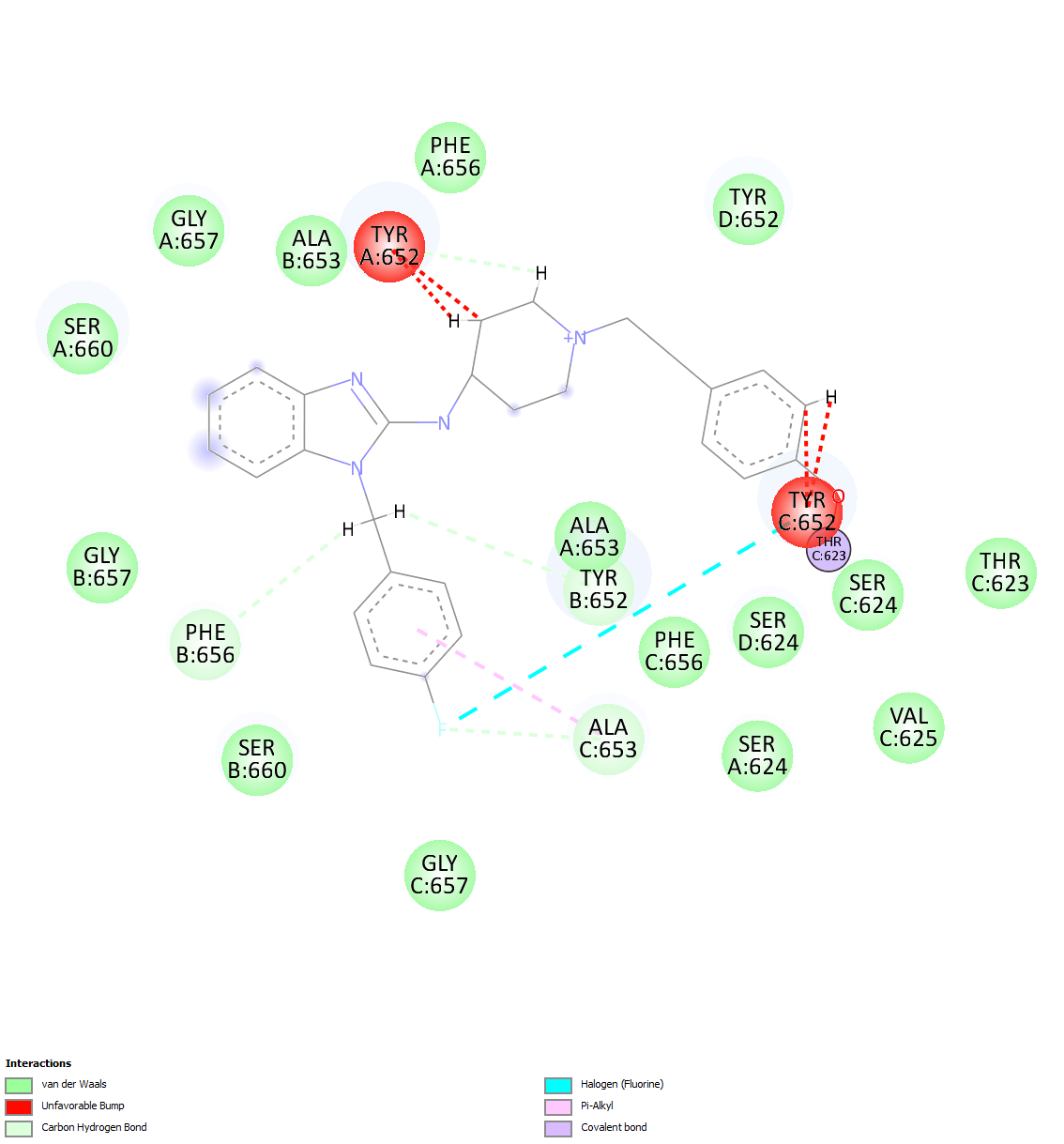

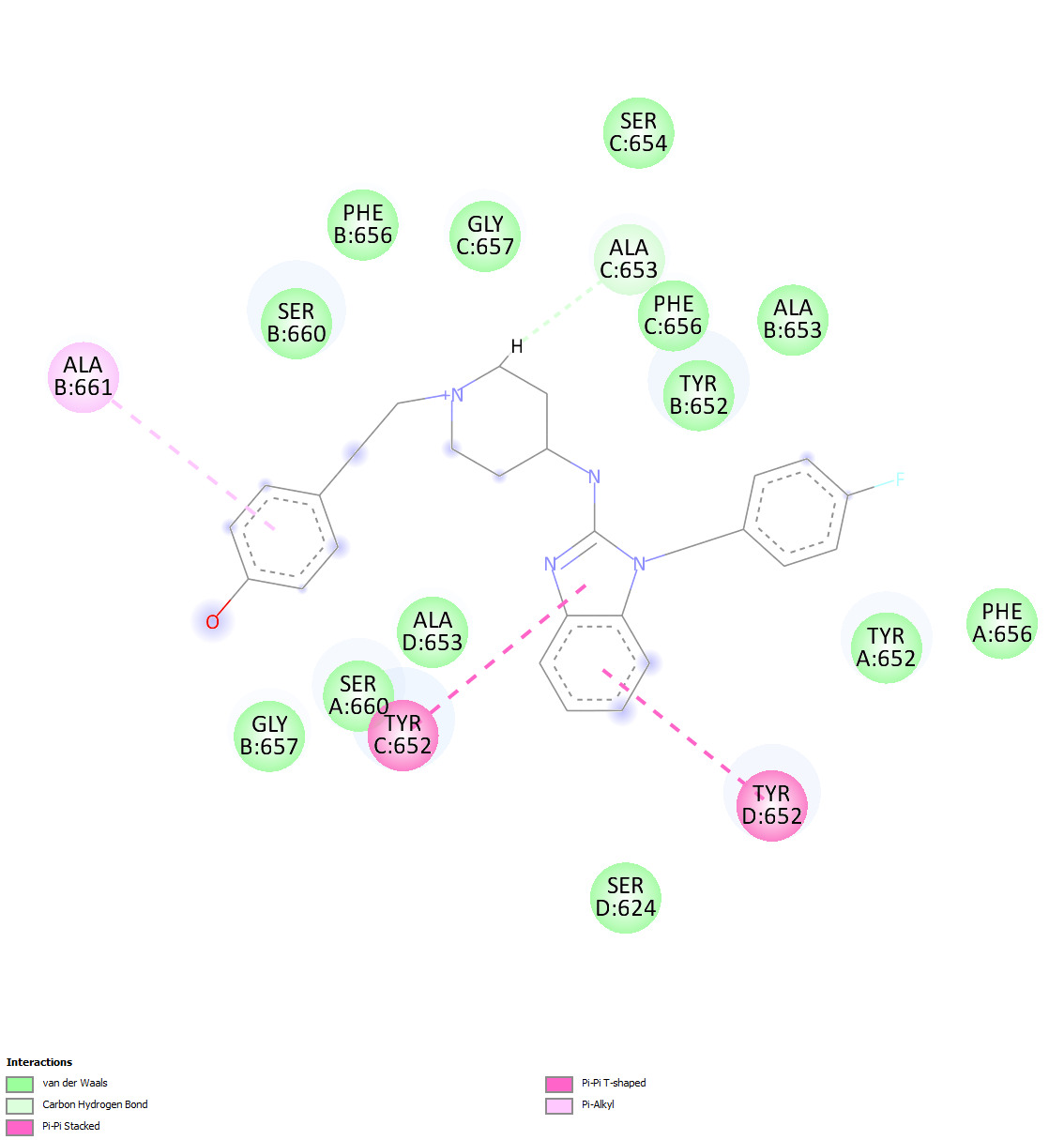


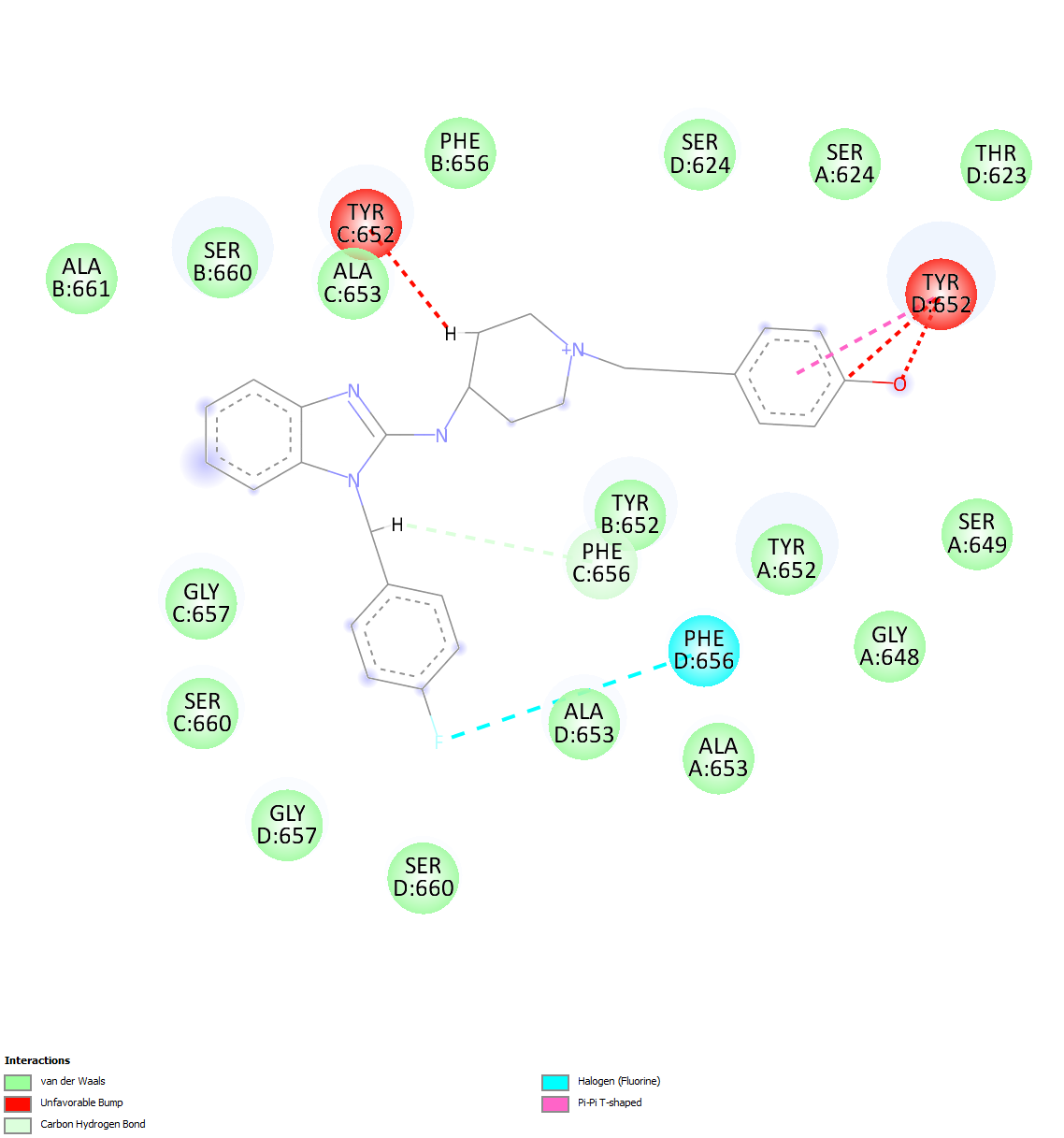

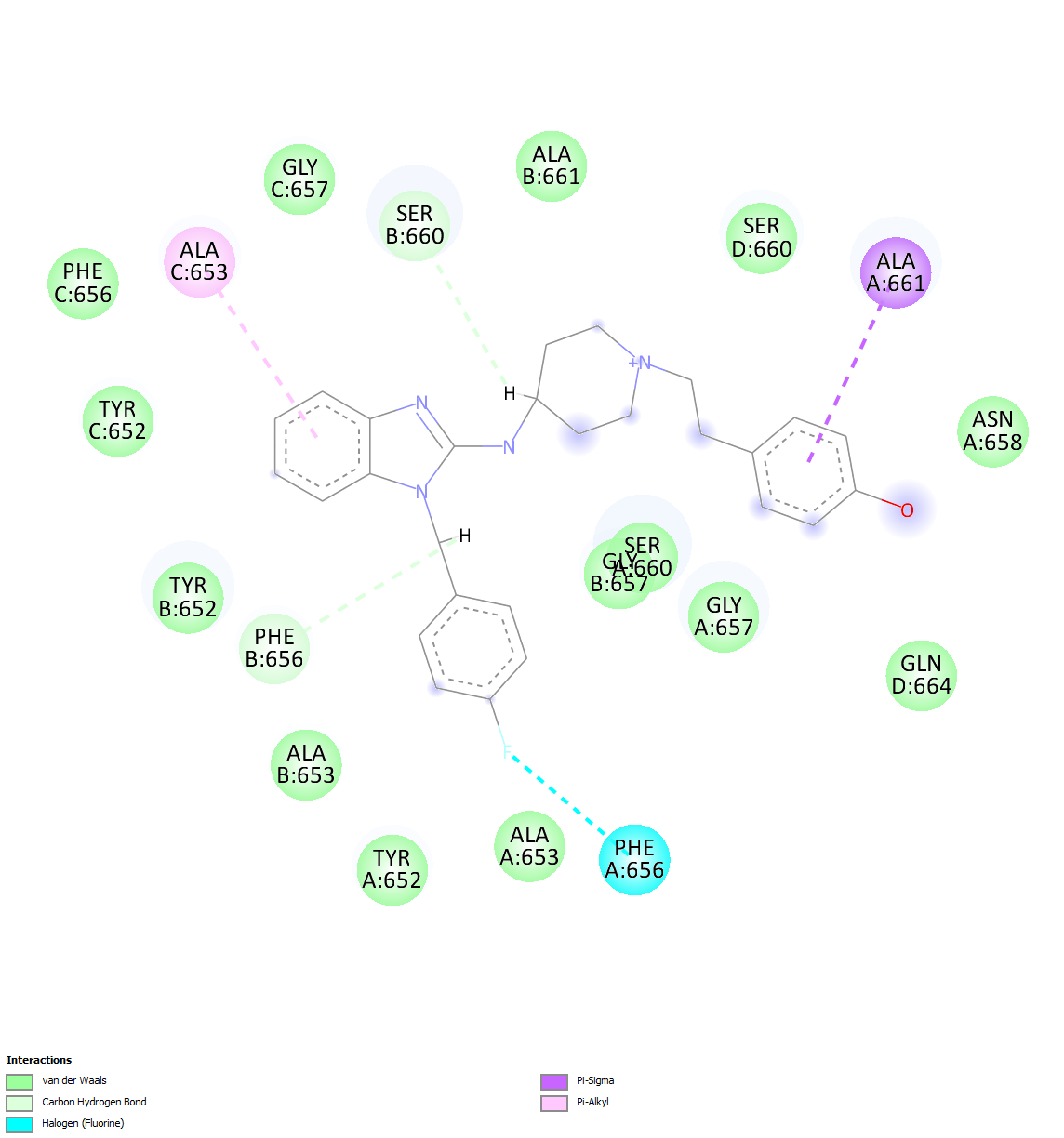


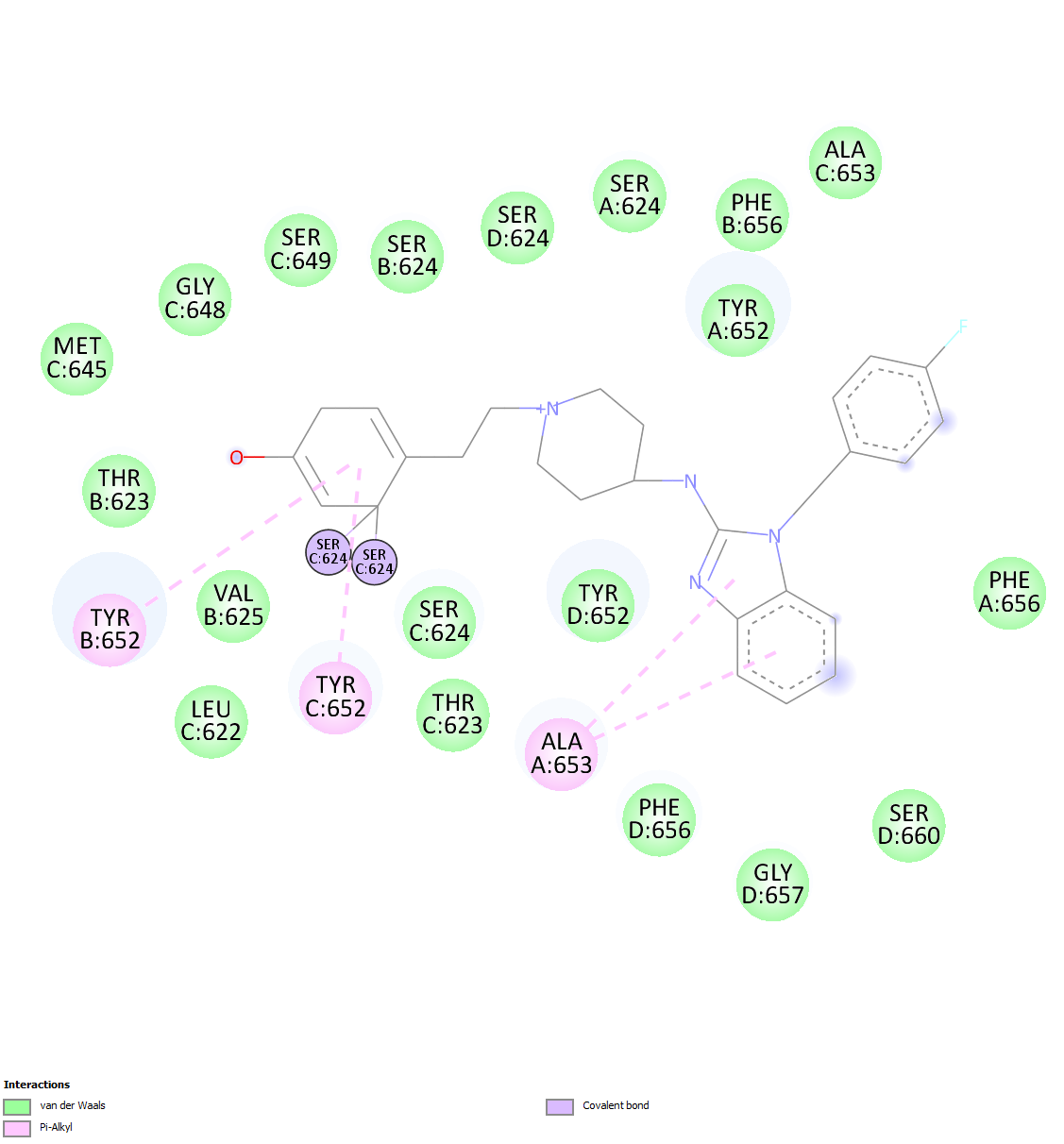

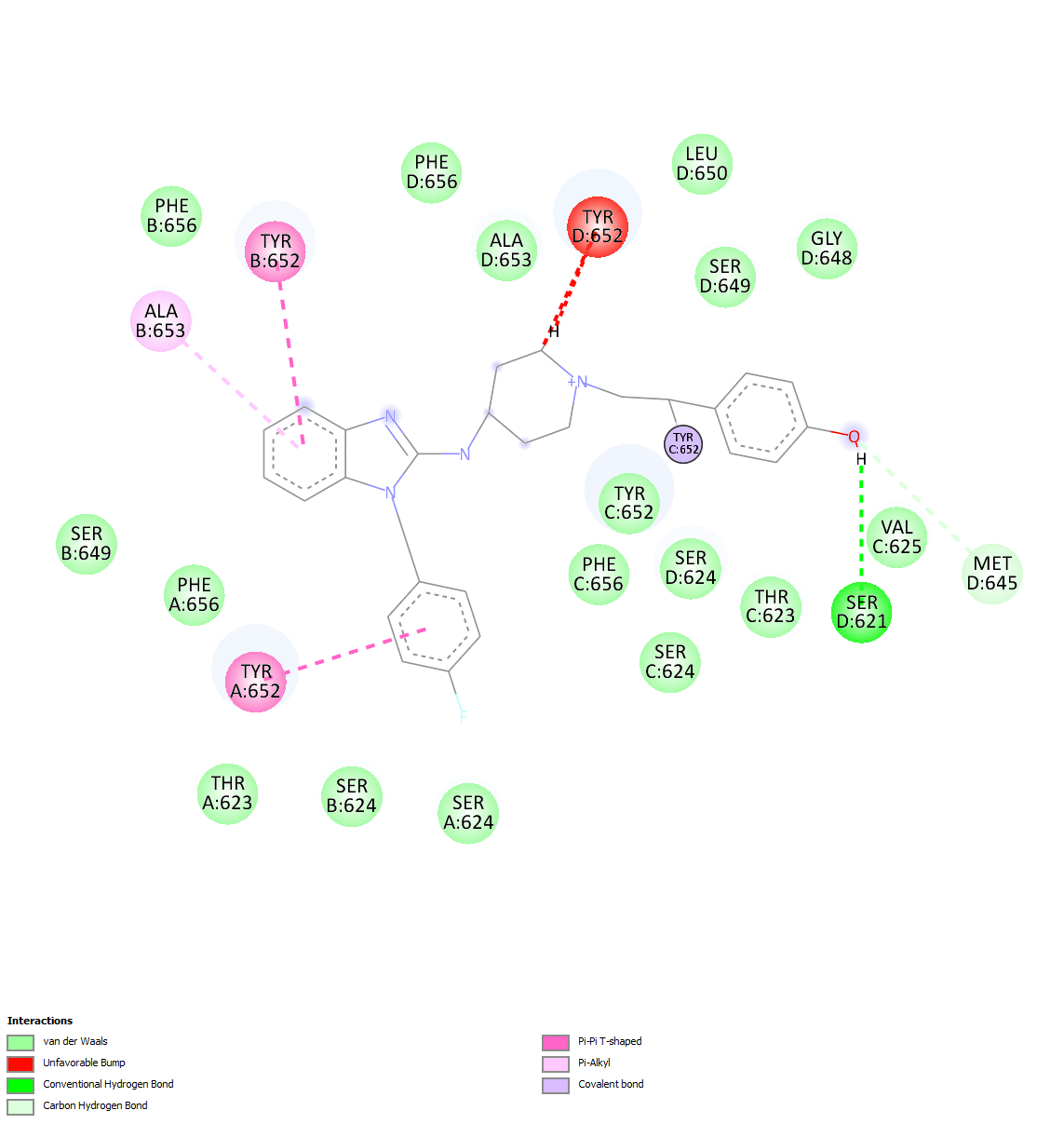


# Figure S7.Complete PatchDock results regarding the top 10 solutions of desmethylastemizole.


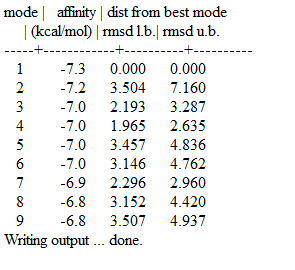


# Figure S8.Complete AutoDock Vina outputs of quetiapine.


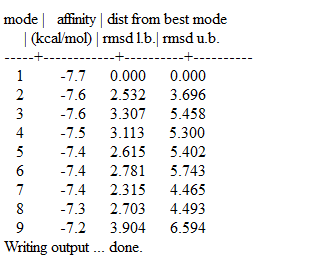


# Figure S9.Complete AutoDock Vina outputs of norquetiapine.


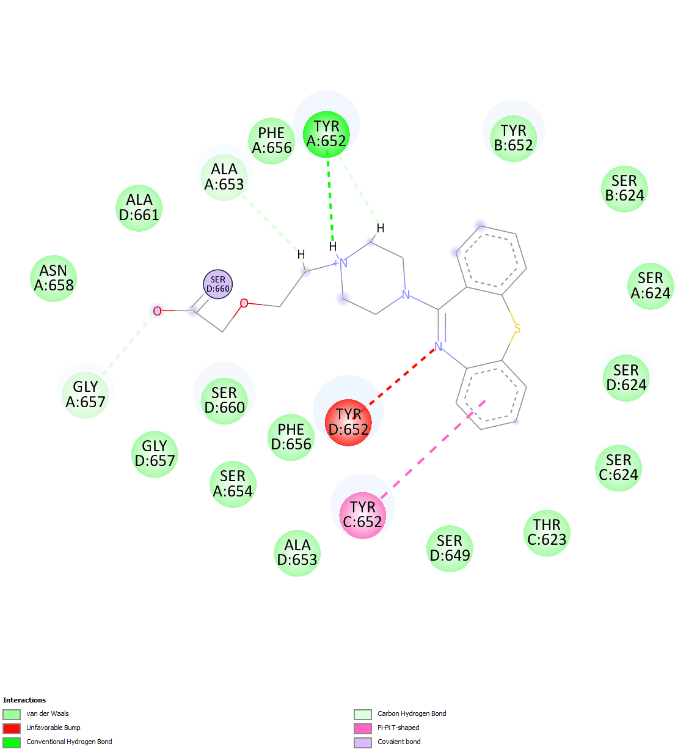

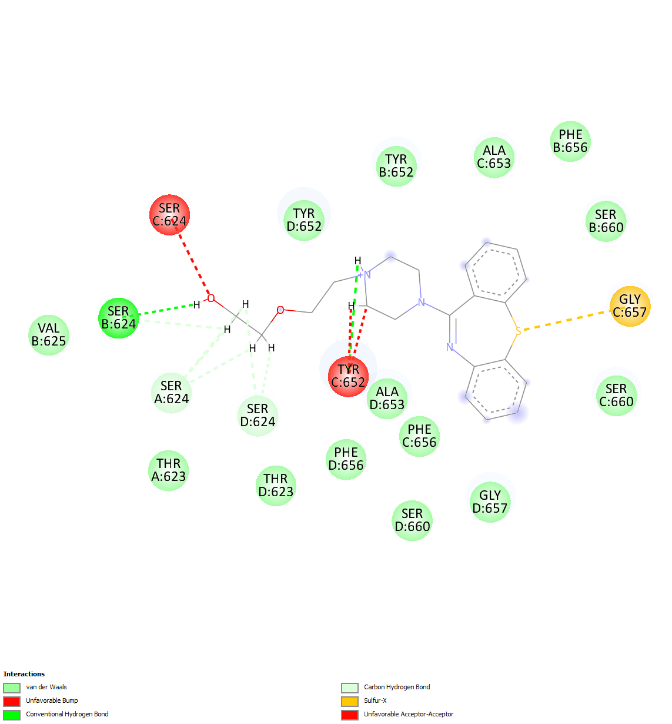


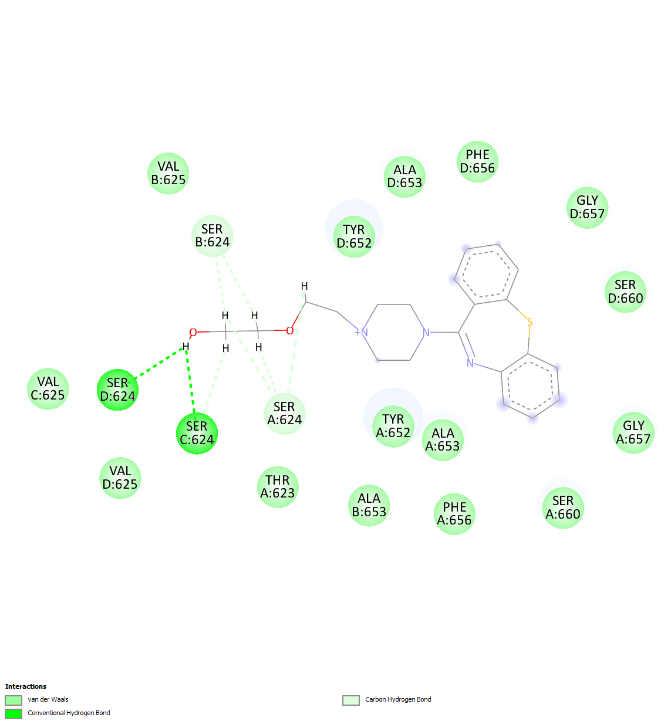

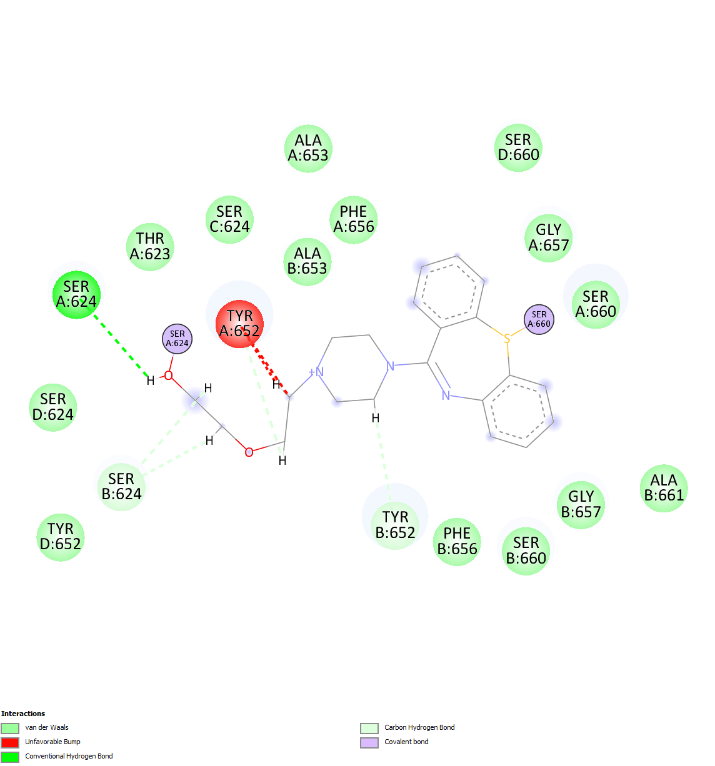


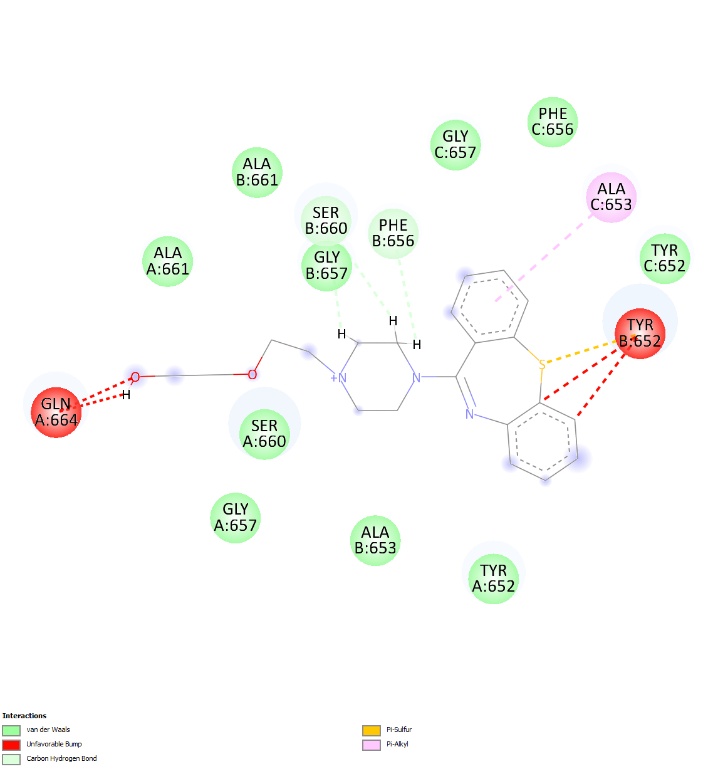

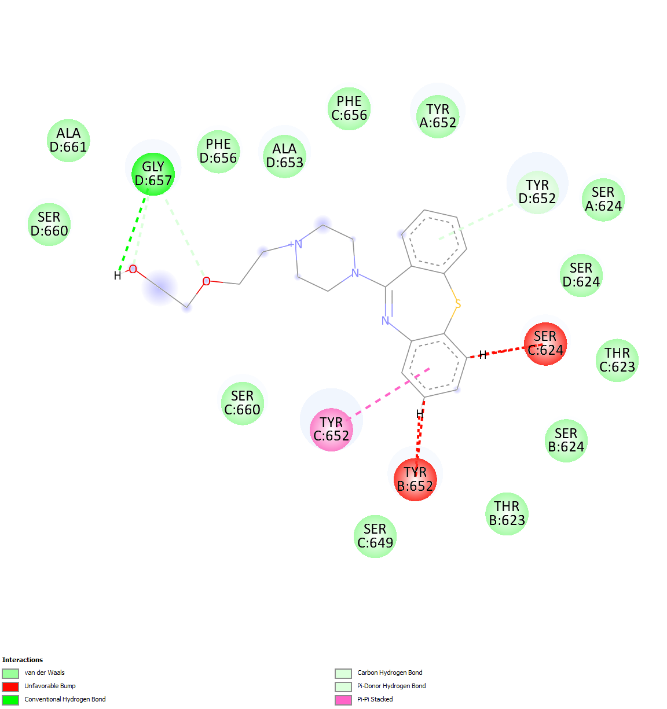


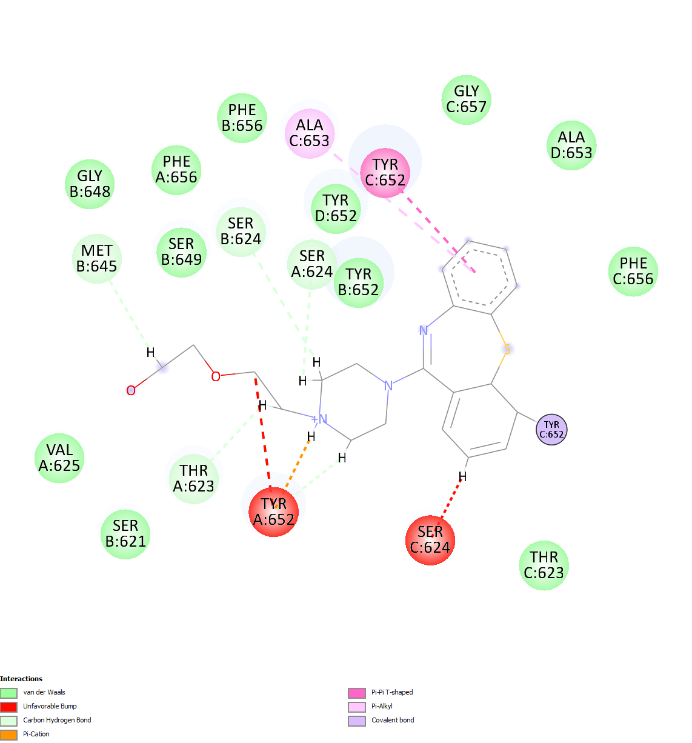

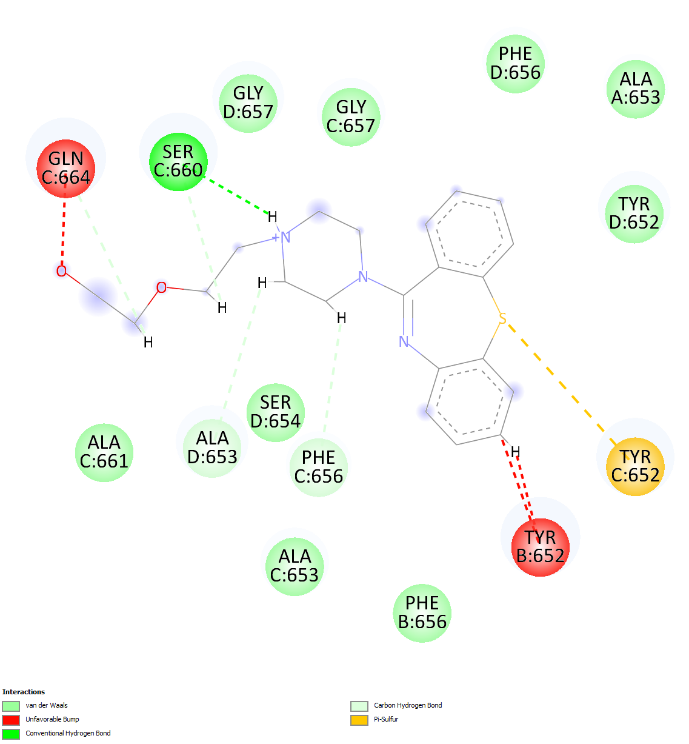


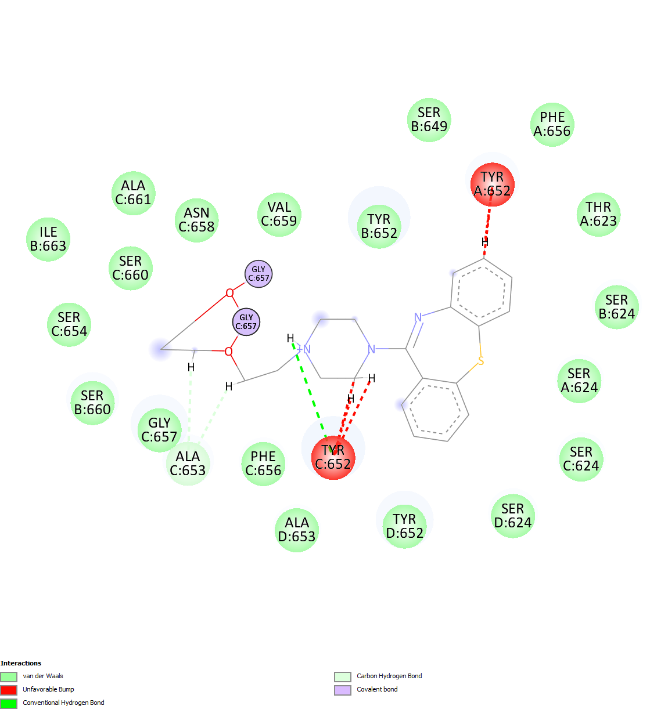

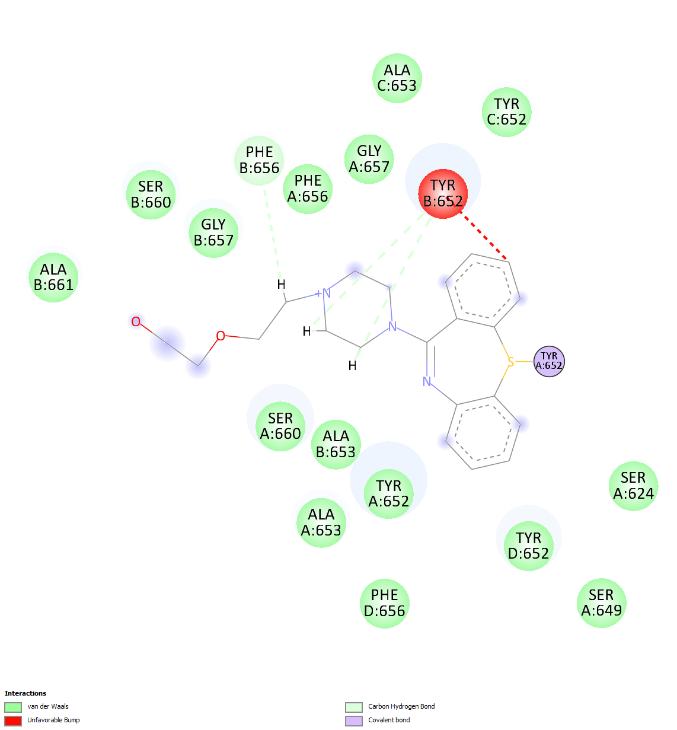


# Figure S10.Complete PatchDock results regarding the top 10 solutions of quetiapine.


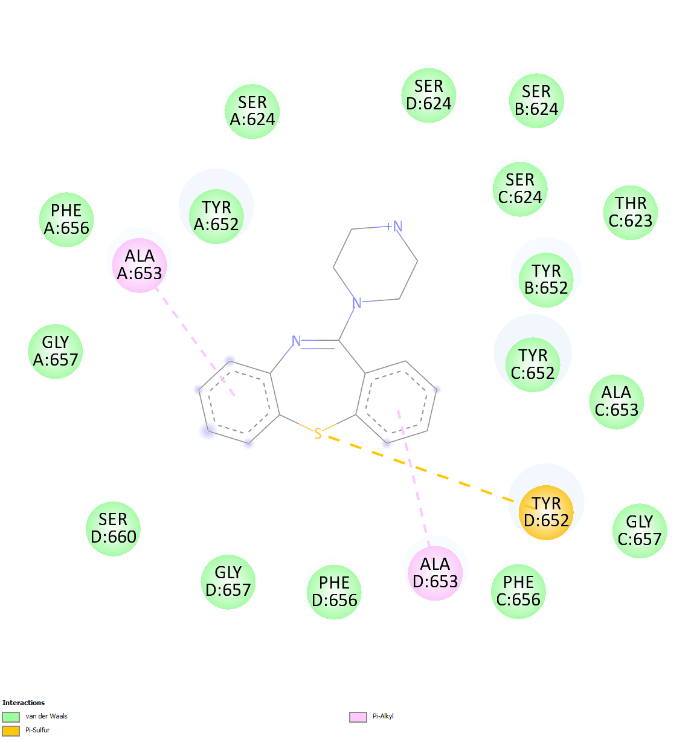

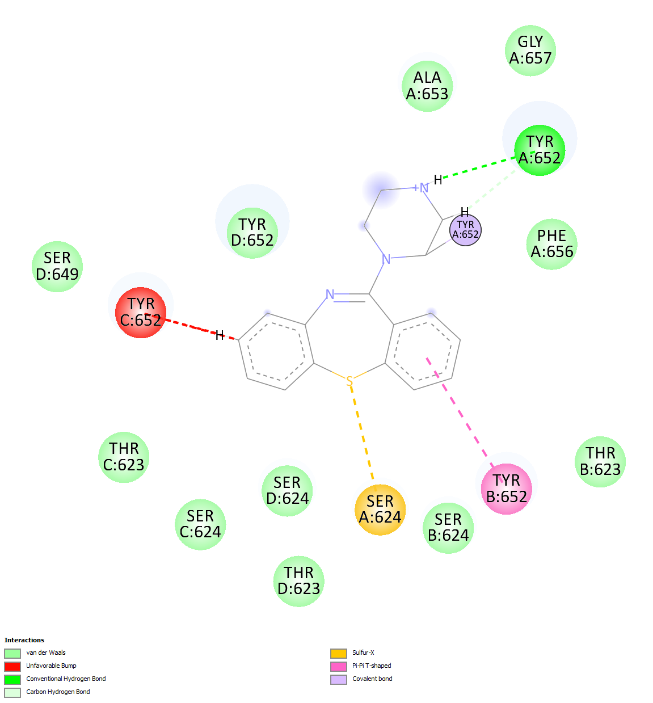


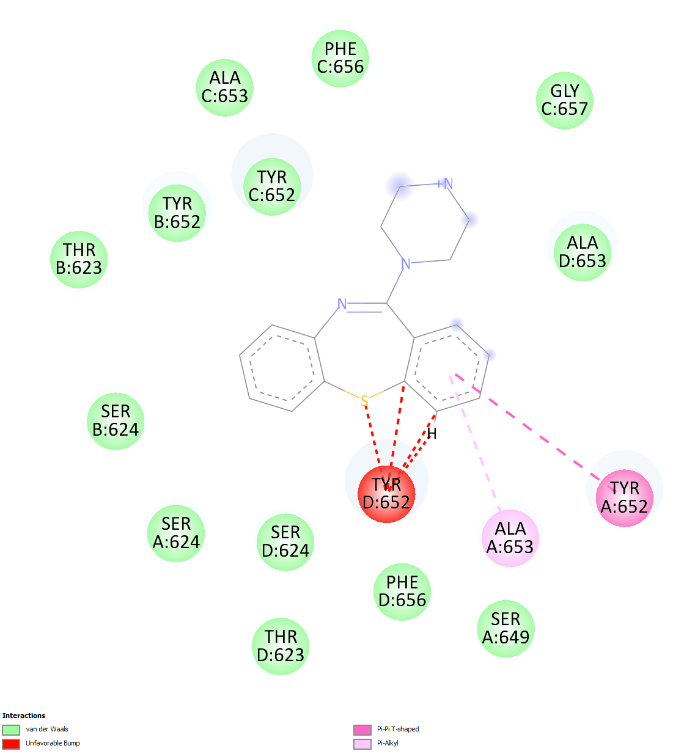

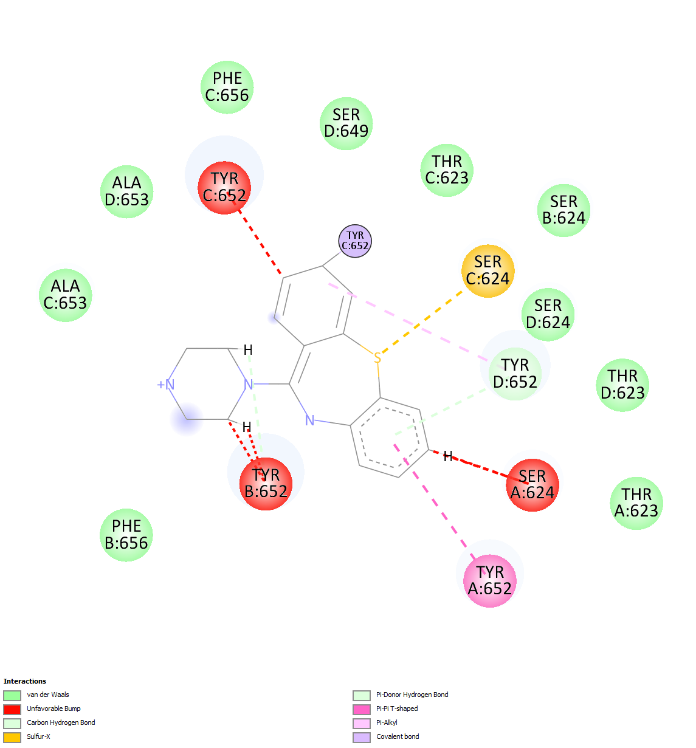


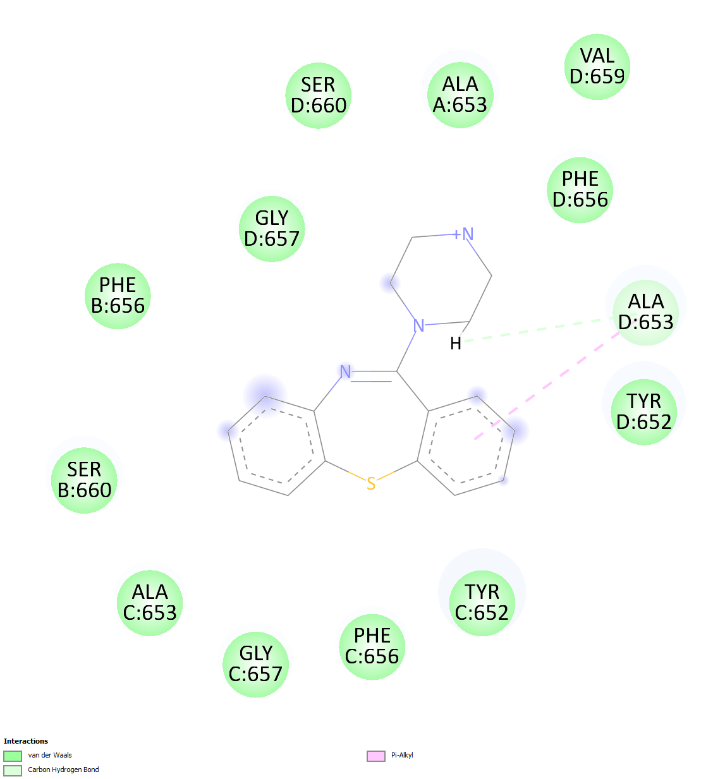

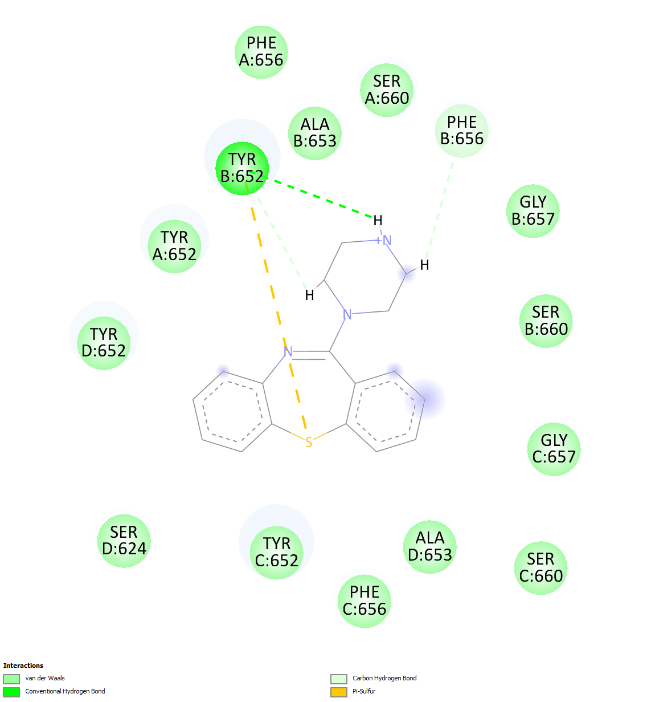


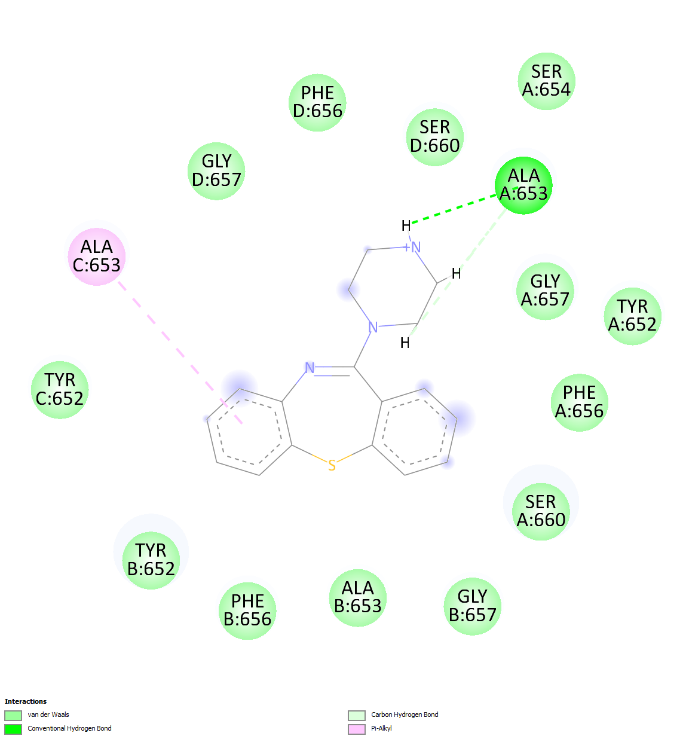

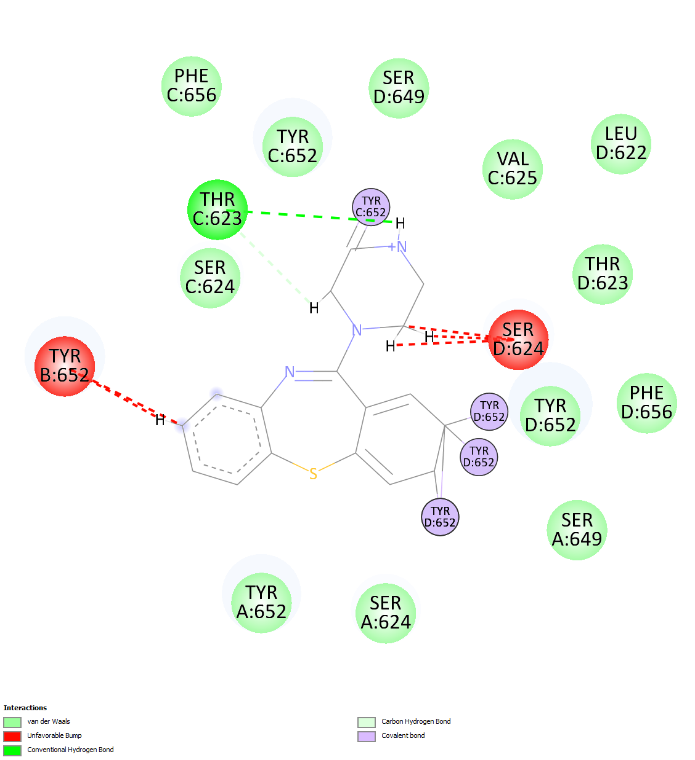


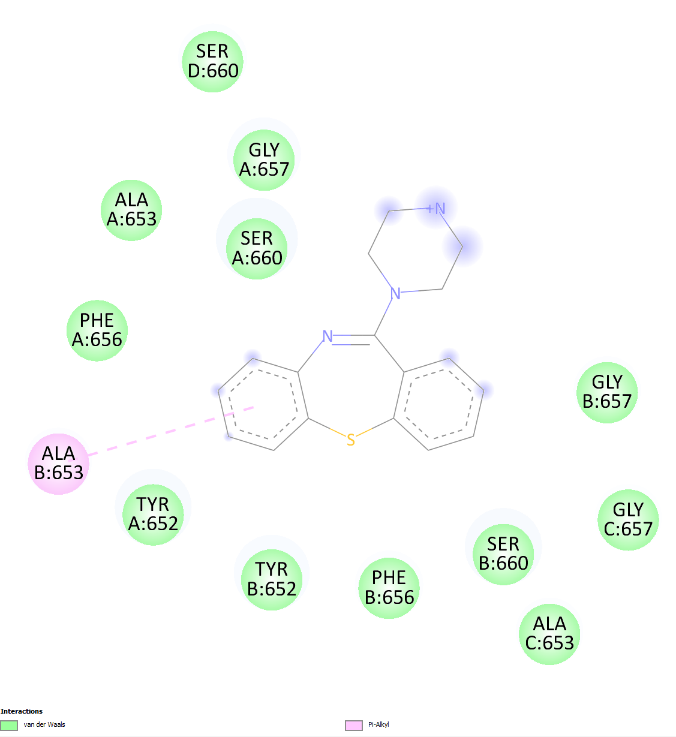

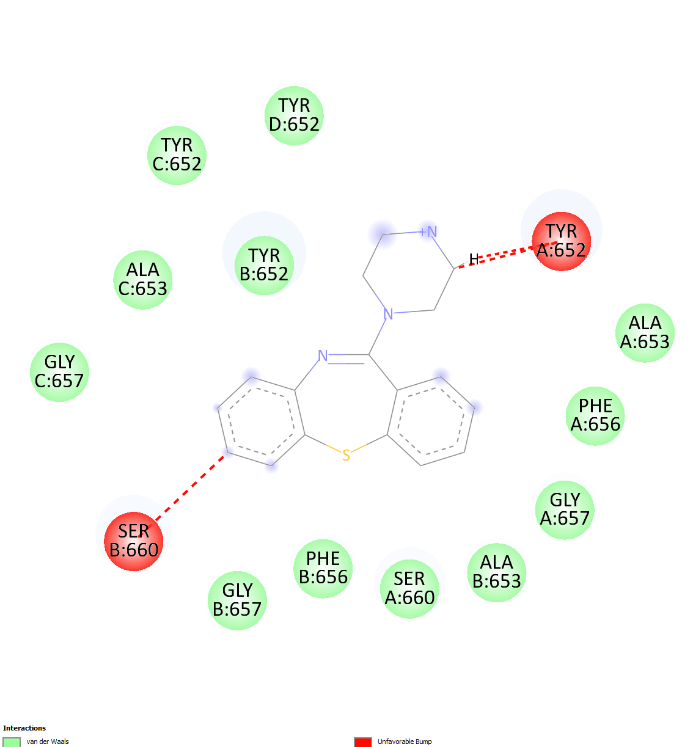


# Figure S11.Complete PatchDock results regarding the top 10 solutions of norquetiapine.


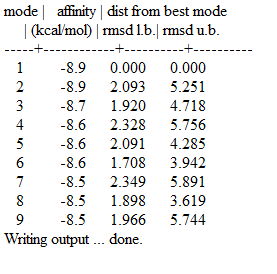


# Figure S12.Complete AutoDock Vina outputs of terfenadine.

#
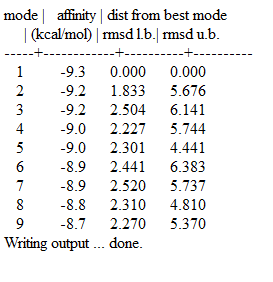


# Figure S13.Complete AutoDock Vina outputs of fexofenadine.


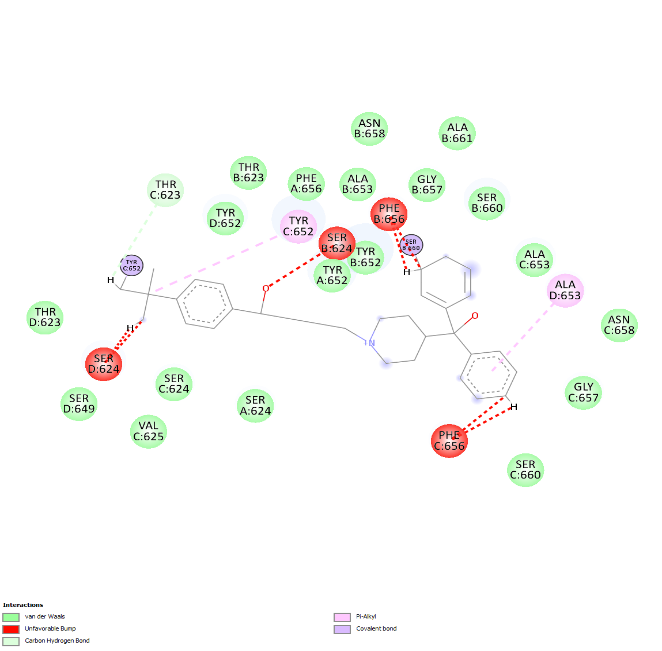

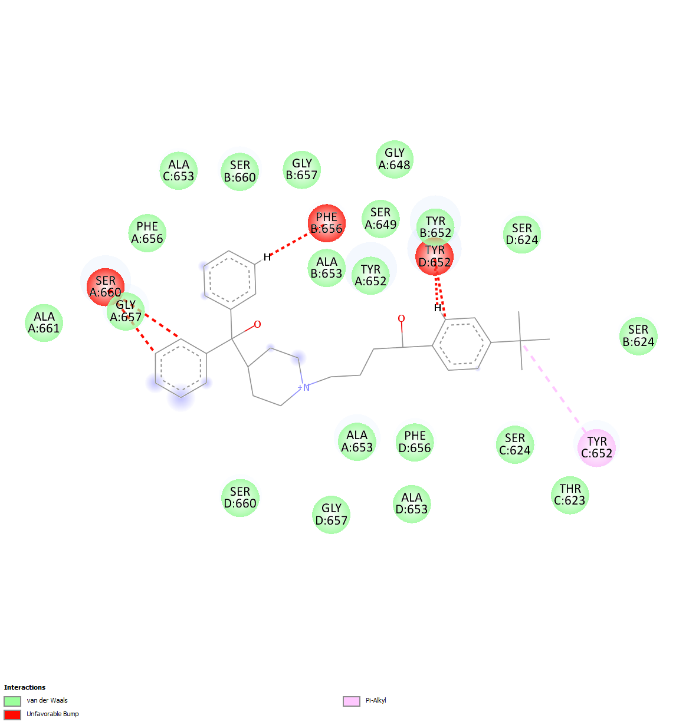


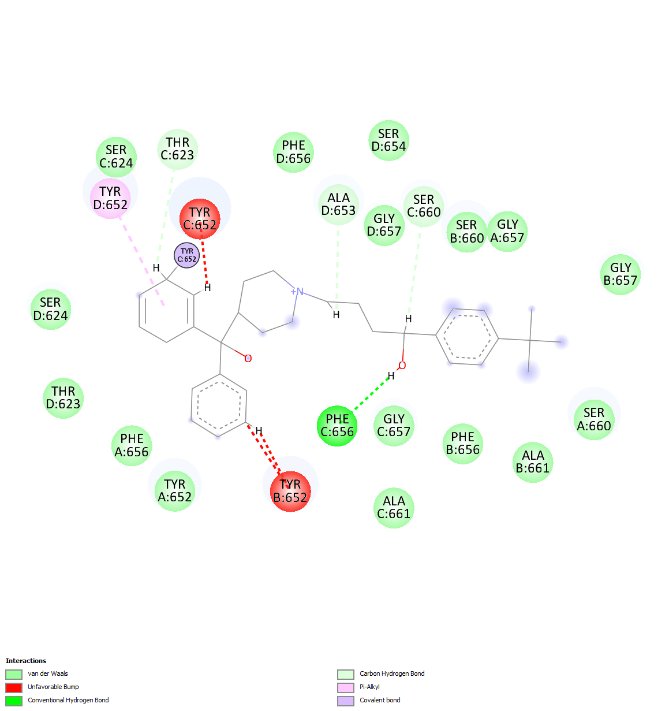

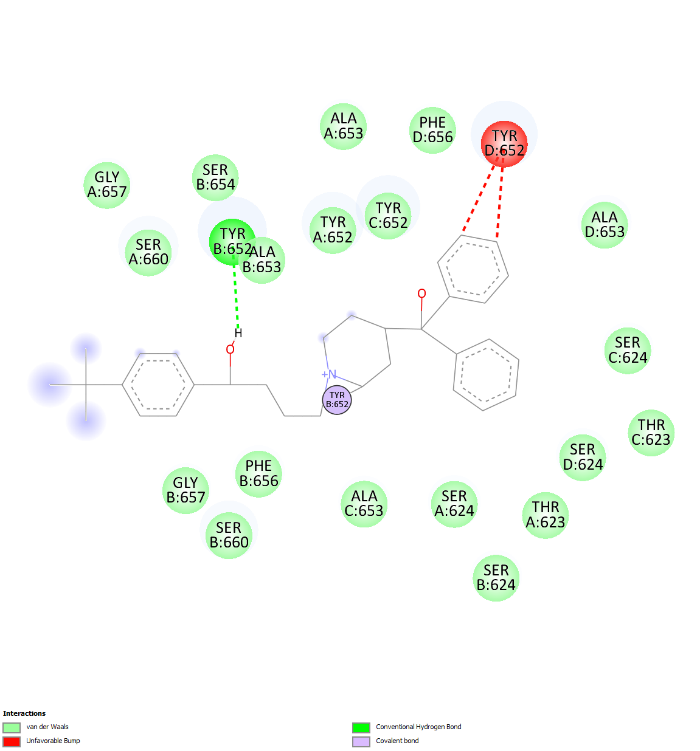


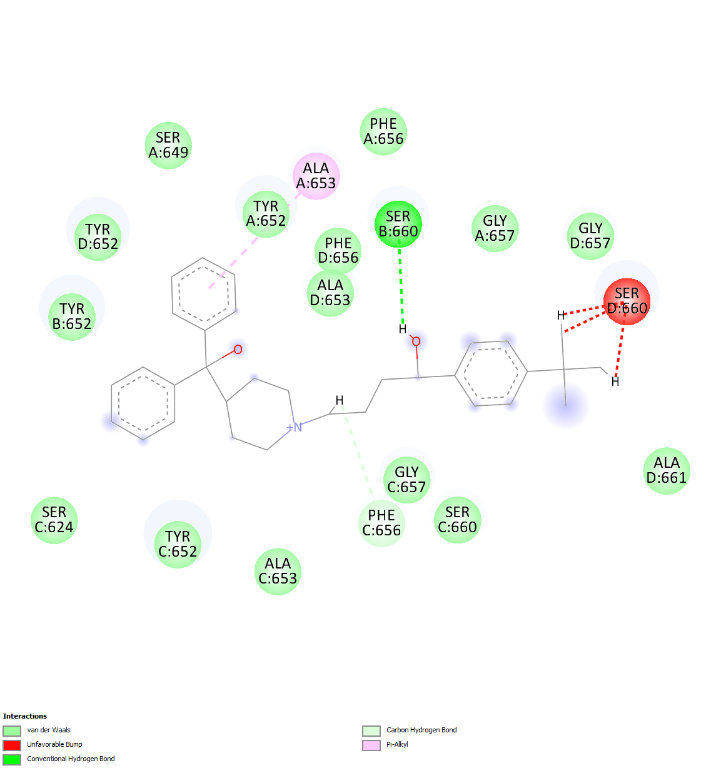

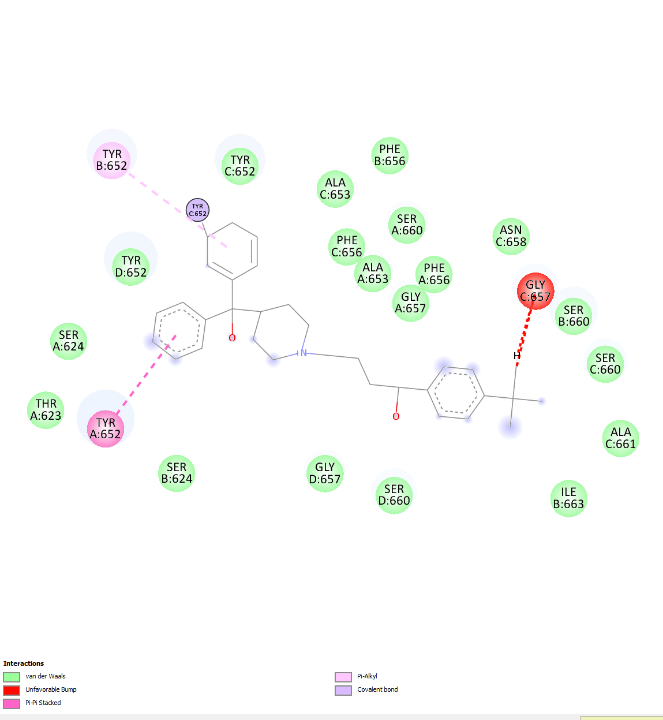


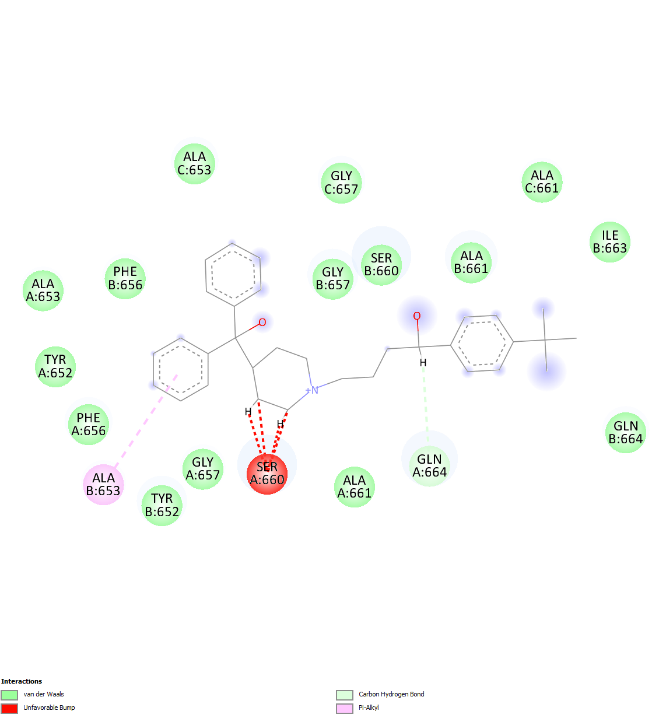

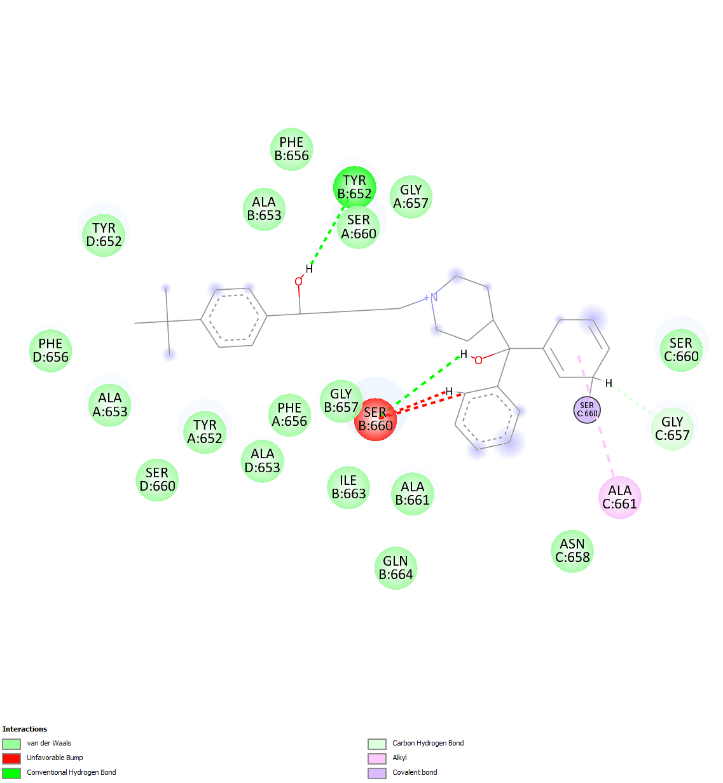


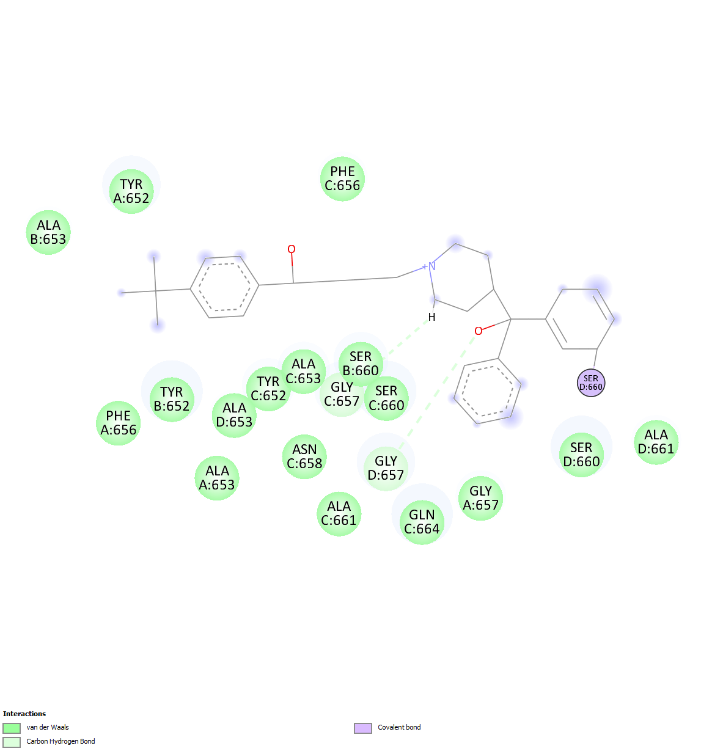

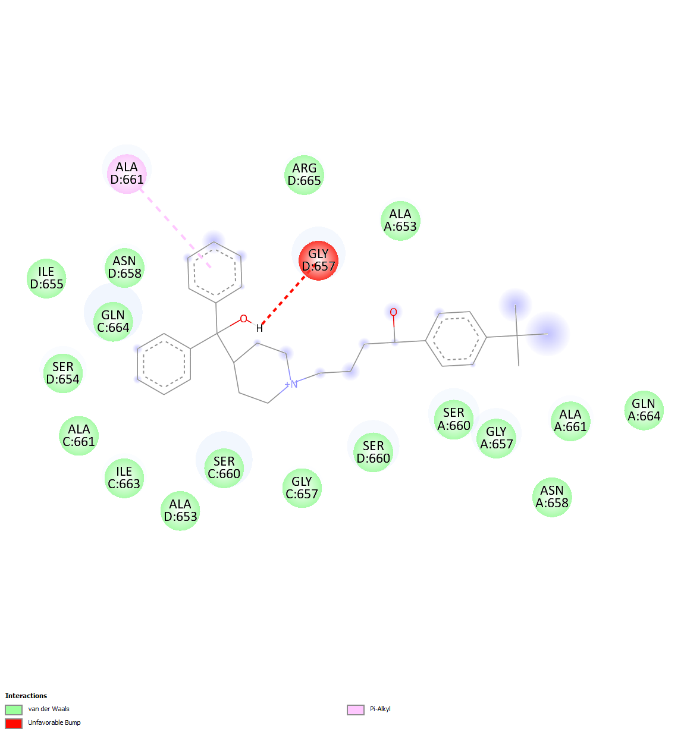


# Figure S14.Complete PatchDock results regarding the top 10 solutions of terfenadine.


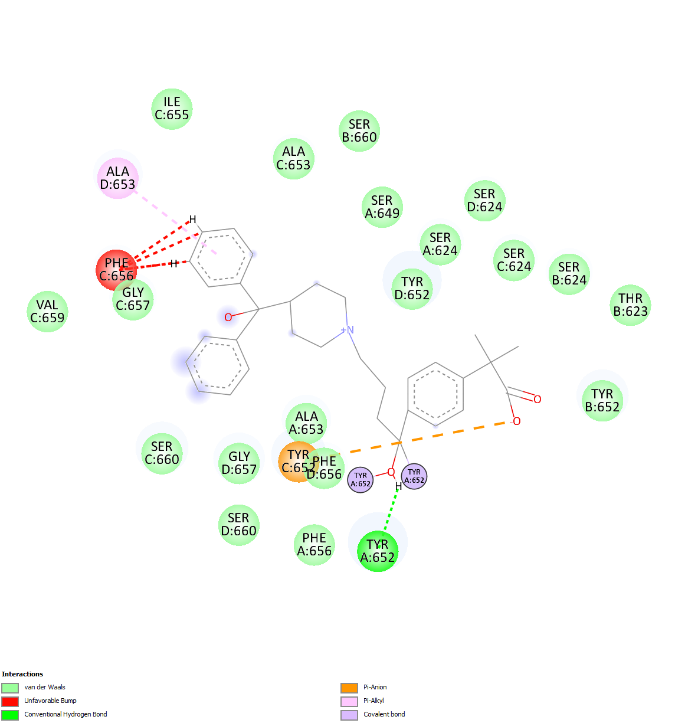

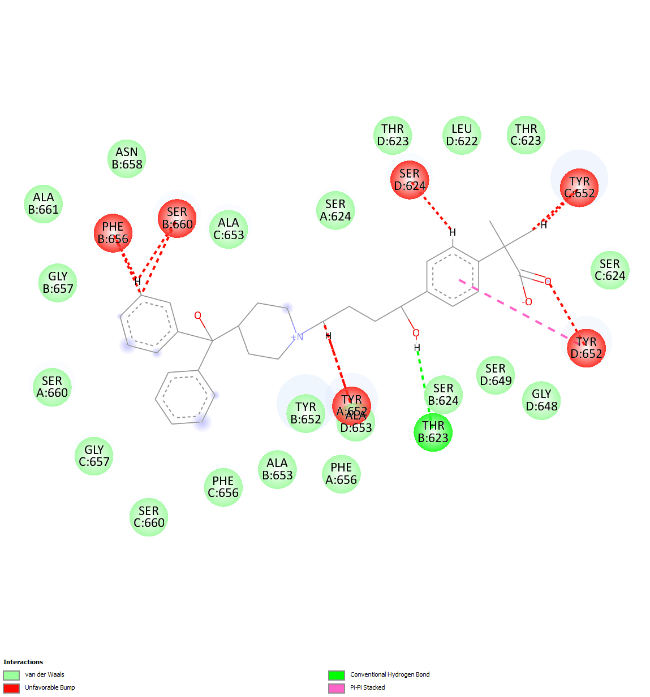


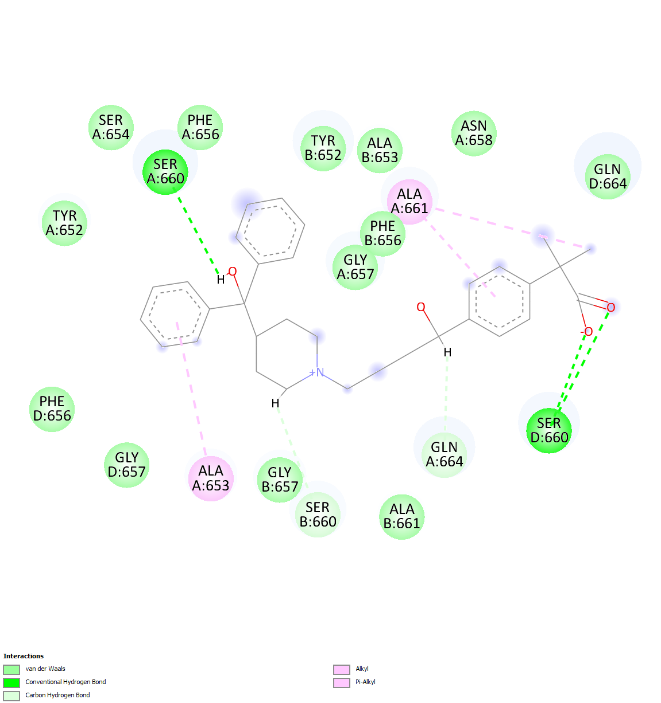

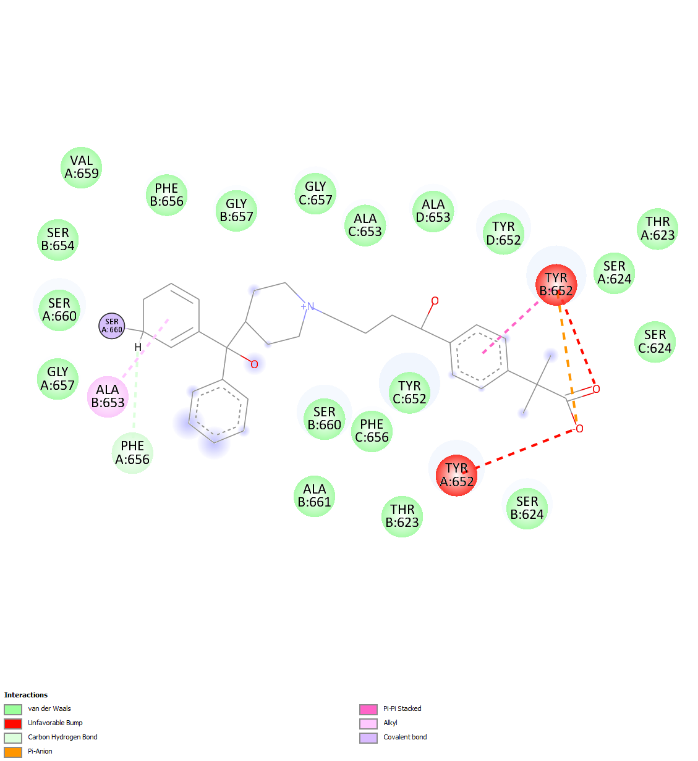


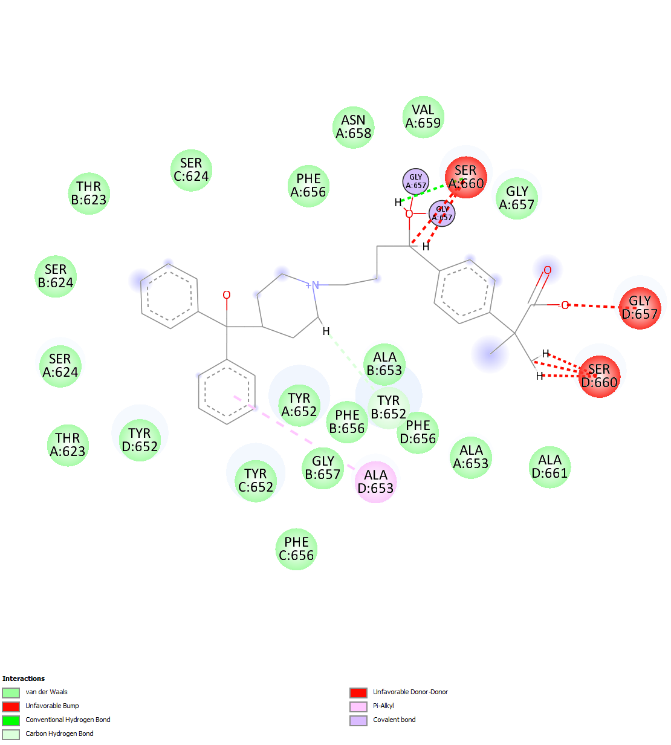

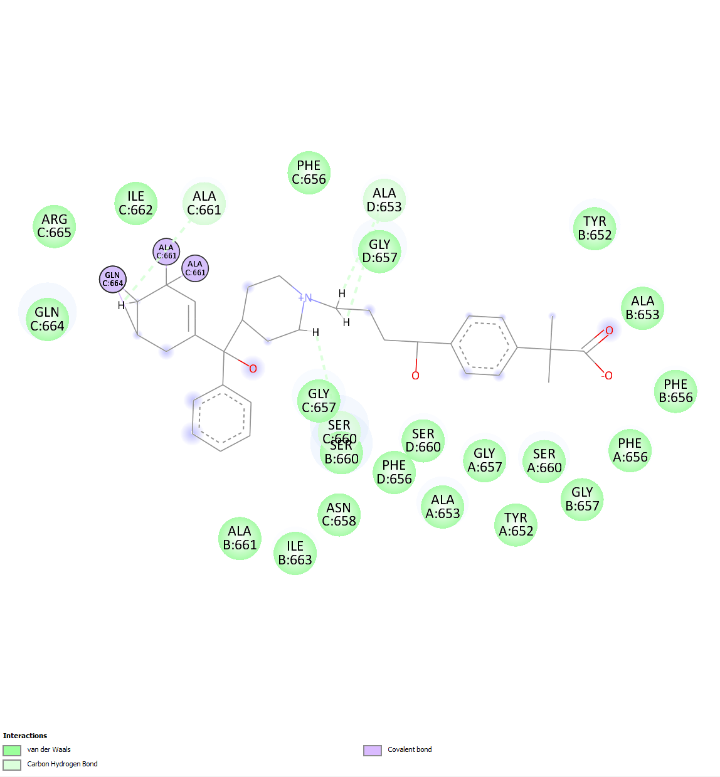


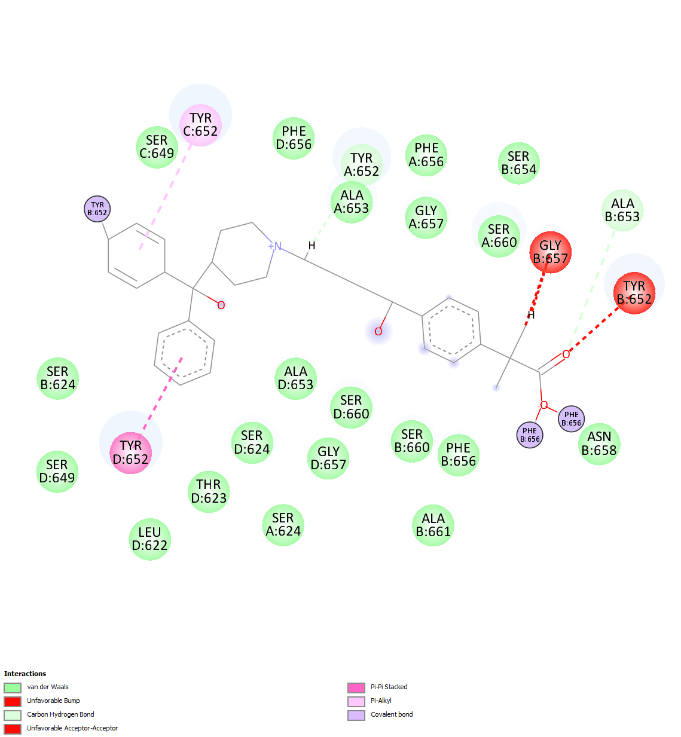

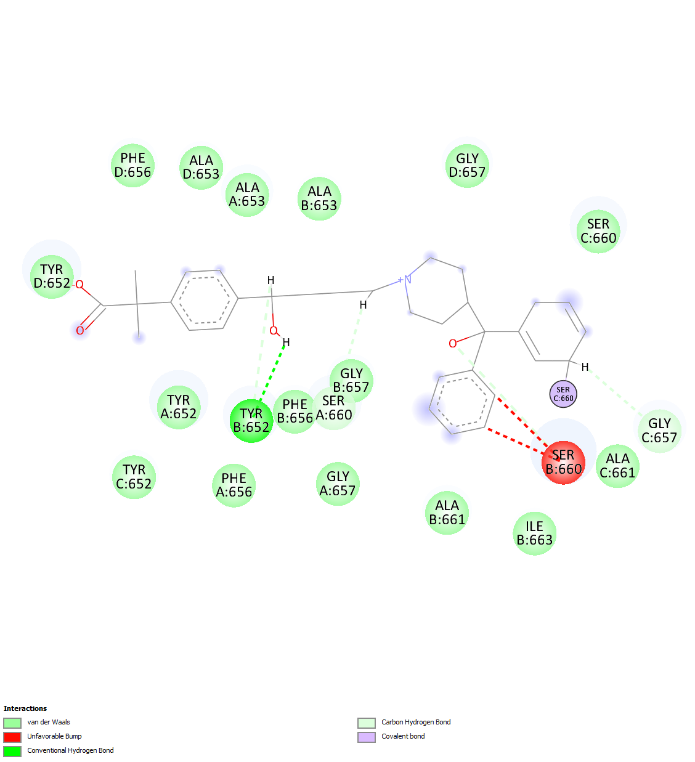


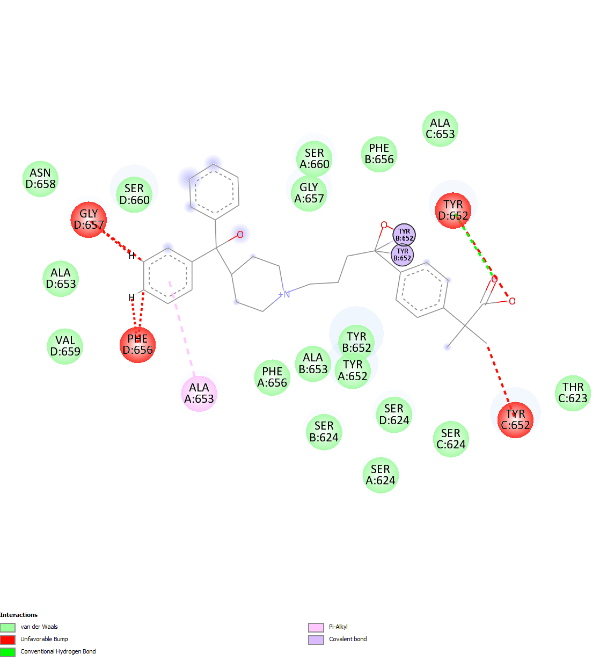

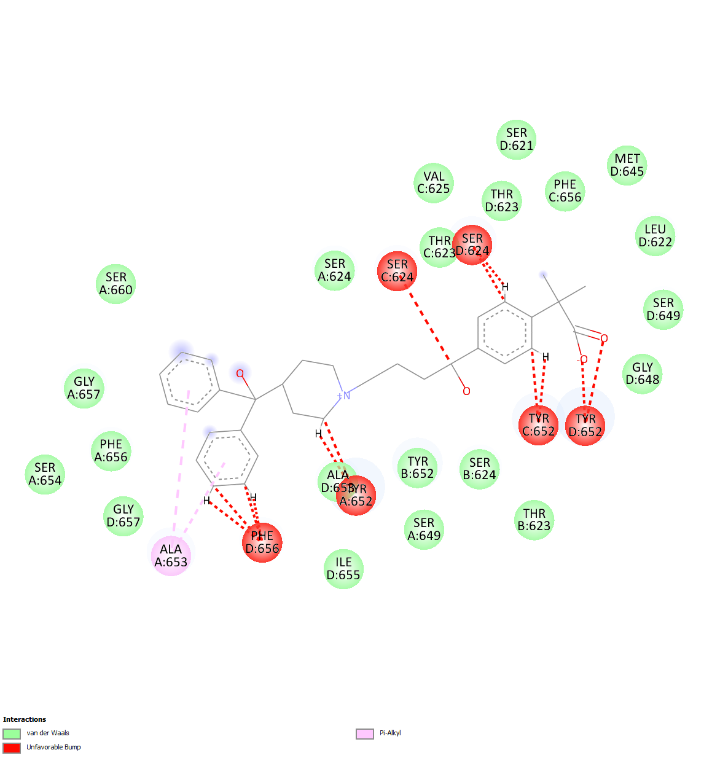


# Figure S15.Complete PatchDock results regarding the top 10 solutions of fexofenadine.
